# Unmet reproductive health needs among women in some West African countries: a systematic review of outcome measures and determinants

**DOI:** 10.1186/s12978-015-0104-x

**Published:** 2016-01-16

**Authors:** Martin Amogre Ayanore, Milena Pavlova, Wim Groot

**Affiliations:** 1 Department of Health Services Research, CAPHRI, Maastricht University Medical Centre, Faculty of Health, Medicine and Life Sciences, Maastricht University, Maastricht, The Netherlands; 2 Top Institute Evidence-Based Education Research (TIER), Maastricht University, Maastricht, The Netherlands

**Keywords:** Unmet needs, Outcome measures, Determinants, Obstetric care, Antenatal care

## Abstract

**Background:**

Identifying relevant measures of women’s reproductive health needs is critical to improve women’s chances of service utilization. The study aims to systematically review and analyze the adequacy of outcome measures and determinants applied in previous studies for assessing women reproductive health needs across West Africa.

**Methods:**

Evidence on outcomes and determinants of unmet reproductive health needs among women of childbearing age in diverse multicultural, religious, and ethnic settings in West African countries was systematically reviewed. The review included recent English language publications (from January 2009 - March 2014). Clinical studies particularly on obstetric care services and reproductive services in relation to HIV/AIDS were excluded. We acknowledge the possibility to have excluded non-English publications and yet-to-be-published articles related to the study aim and objectives. Outcomes and determinants were assessed and defined at three main levels; contraceptive use, obstetric care, and antenatal care utilization.

**Results:**

Results show increasing unmet need for women’s reproductive health needs. Socio-cultural norms and practices resulting in discontinuation of service use, economic constraints, travel distance to access services and low education levels of women were found to be key predictors of service utilization for contraception, antenatal and obstetric care services. Outcome measures were mainly assessed based on service utilization, satisfaction, cost, and quality of services available as core measures across the three levels assessed in this review.

**Conclusions:**

Evidence from this review indicates that currently applied measures of women’s reproductive health needs might be inadequate in attaining best maternal outcomes since they appear rather broad. More support and research for developing and advancing context-related measures may help to improve women’s maternal health.

**Electronic supplementary material:**

The online version of this article (doi:10.1186/s12978-015-0104-x) contains supplementary material, which is available to authorized users.

## Background

Globally, more than half a million women aged 15–49 years die annually from preventable pregnancy-related complications [1, 2]. Women in developing countries have a 1 in 26 chance of dying from pregnancy and abortion compared to 1 in 9400 chances in Europe [3]. One of the factors associated with this outcome is the unmet health need for contraception and reproductive health services. Unmet reproductive health needs exist if there is a gap between a perceived need and the current available options to satisfy the need. This paper focuses on three levels of unmet reproductive health needs: contraceptive service use, obstetric care, and antenatal care utilization.

Contraceptive prevalence rate (CPR) globally for women aged 15–49 years, married or in a union rose from 58.4 % in 1994 to 63.6 % in 2012. Yet, an estimated 222 million women still fall within the current unmet needs estimates for contraceptives, with 90 % of these women currently in the developing world [2, 4, 5]. As a result, one in five pregnancies is unintended [6].

Women often resort to an abortion when faced with an unintended pregnancy [4]. These women are said to have an unmet need for contraception. Regarding antenatal care utilization, an estimated 122 million women in developing regions needed antenatal and postnatal care in 2012 but only 55 % of these received four or more antenatal visits [7]. But it is also evidenced that the unmet need for antenatal care decreased in developing countries in the period 2000-2012 [8].

Thus, despite the global health problems, family planning and antenatal service uptake have improved over the decades in developing countries [9].

Nevertheless, more effort is needed to change the results in Sub-Saharan Africa (SSA). SSA is evidenced to have the highest percentage of women with unmet reproductive health needs (approximately one out of every four women) [10]. Also, SSA accounts for 24 % of the global disease burden whiles only 3 % of the world skilled health workforce come from this region [11]. As evidenced in the literature, long travel distances to the nearest health facility, poor access and poor quality of maternal and newborn care provide a low probability of mother and child survival especially among rural women in SSA [12].

This paper addresses the unmet reproductive health needs in West Africa as this region has one of the poorest maternal and reproductive health indices in SSA [2]. The situation is even worsening with an ever surge in population growth over the last decade [3]. In 2012 alone, 222 million women in developing countries were reported to have unmet need for modern contraception [13]. This level of unmet need decreased in every sub-region between 2003 and 2012, but remains high in West Africa (74 %) compared to East Africa (54 %) [11]. Additionally, West Africa has lower levels of modern contraceptive use (≤26 %) compared with the higher levels of use (46–66 %) recorded in East Africa and Middle Africa respectively [11]. Since the review covers the West African countries, context-related similarities and differences should be acknowledged. Population policies to reverse the rising population growth rates have existed for the past decades in all countries in West Africa [14–16]. However, much remains to be improved on ripping the demographic dividends of fertility declines in these countries [16]. Study reviews in across West Africa show low uptake of contraceptives due to reported contraceptive failures [11, 17] whiles religion, gendered interest, and social parity desires have also been long documented to work against family planning interventions [18–20]. The situation of reproductive health services is especially challenging in countries recovering from civil war and instability, such as Senegal, Sierra Leone among others, while countries with political stability and continual economic growth, like Burkina Faso, Ghana and Nigeria, have achieved political commitment and progress in improving these services. Comparative evidence provided by the United Family Planning Population Agency (UNFPA, 2010) show that Benin, Burkina Faso, Mali, Nigeria, and Senegal witnessed significant positive changes in the levels of unmet needs over the last decade [17]. For example, Nigeria recorded a high percentage point change (19.5 %) between 2003 and 2008 for unmet need levels. Burkina Faso, Benin and Mali recorded percent changes of 11.6 % (1999–2003), 9.9 % (2001–2006) and 9.5 % (2001–2006) respectively. However, no country was evidenced to have significantly improved its contraceptive use rates, although Burkina Faso and Nigeria were estimated to have made few positive changes on contraceptive prevalence levels. Burkina Faso recorded a percentage point change of 16 % from 1999 to 2003 whiles Nigeria is evidenced to have a 15.9 % improvement between 2003 and 2008 [17]. Overall, in West Africa, individual context factors such as gender and social roles interact and shape reproductive health behaviors across all population groups [19–22]. Wealth, education, residence type, and age disparities however remain the greatest influencing factors introducing disparity in any context [11, 17, 23]. Overall, policy implementations aimed at improving reproductive health outcomes vary from country to country depending on the level of accountability and transparency, economic growth, good governance principles, and social capital of the citizenry [11, 17]. The fundamental recognition is that no single policy intervention is sufficient in any country context to make huge improvements in maternal reproductive health outcomes. The identification of outcome measures and determinants of women’s reproductive health needs, which is the focus of this review, may help improve broader consensus on women’s reproductive needs for greater health and economic outcomes.

This paper assesses outcome measures and determinants used in previous studies to provide evidence on the adequacy of outcome measures and determinants applied in previous studies in relation to unmet reproductive health needs. This can be used to address policy and research gaps related to maternal health in this region. No such comprehensive review has been undertaken in West Africa. The review will provide further impetus for redoubling of commitments towards improving maternal health outcomes in SSA.

## Methods

**Fig. 1:**
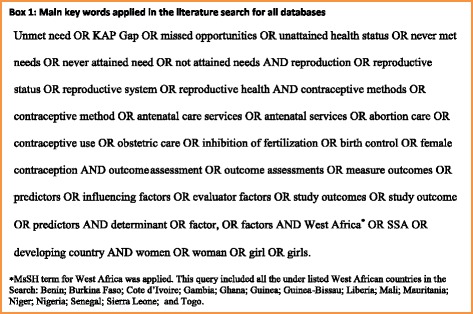
Main chain of key words applied in the literature search across seven databases. Possible synonyms and MeSH term categories for all key words were developed and applied in all database searches

To assess outcome measures and determinants of unmet reproductive health needs among women in West Africa, a systematic review of recent publications in the area was carried out in 2014 following the Cochrane review protocols [24]. The following key words were applied in the search: unmet needs, reproductive health, contraception, contraceptive use, contraceptive service, outcome measures, determinants, abortion care, antenatal care, West Africa and women. A large number of possible synonyms were generated for the main key words. These synonyms were then included in the extensive search across seven databases to identify relevant papers for this review (see Fig. 1). The seven databases included PubMed, Econpapers, CINAHL, Psych INFO, Science Direct, Embase and Biomed. The objective was to establish any scientific work in relation to the review. In the PubMed search, MsSH term categories were applied across all key words to ensure that all relevant publications were extracted for the review. A full list of the main key words and MsSH word categories used in the search can be found in Fig. 1. The names of the 15 Western African countries were included in the MESH term categories. Separate search per country was also carried out. The following inclusion criteria were used in the search. Publications that were published only within the last five years (January, 2009- March, 2014) were included. When publications reported the same subject matter, the most recent one was assessed. All publications outside the scope of this review were excluded from the review. The publication list was subsequently filtered to cater for only English language publications focused on women and within the boundaries of West Africa. Papers on reproductive health issues that were included defined outcome measures and determinants at three main broad indicator levels; contraceptive use, abortion and antenatal care services. All other issues of reproductive health outside this spectrum were excluded from the review. Clinical studies particularly on obstetric care services and reproductive services in relation to HIV/AIDS were excluded from the review.

The reference lists of the publications were also reviewed and those found to meet the review criteria were added to the list of reviewed publications. The quality of the publications included in the review was assessed. For country publications with more than one estimate for the outcome measures, the national estimates provided by the studies based on the Demographic Health Surveys (DHS) were used. Where national DHS data were not available, and more than one value was reported for the outcome measures at a national level, an average estimate was calculated. Local level estimates were not applied in this review. Thus, all outcome measures for unmet needs in this review were based either on DHS data or national level data between 1997 and 2008. Review results were obtained and presented in the form of tables. The results section elaborates further details of these results. Figure 1 illustrates the flow of search results as prescribed by the PRISMA Group for systematic reviews and Meta-Analyses [24].

## Results

**Fig. 2:**
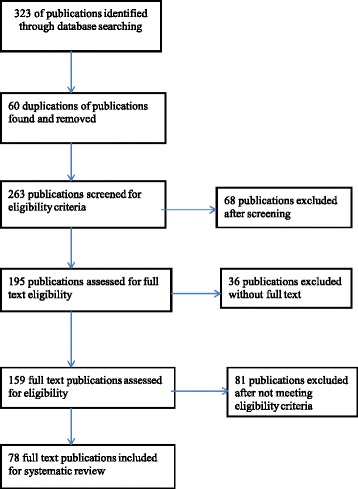
Flow diagram of systematic review process. This flow applies the principles of PRISMA 2009 flow diagram for systematic reviews

A total of 323 publications were identified during the systematic search. This initial list included publications for 14 West African countries: Nigeria (103), Ghana (96), Senegal (23), Benin (17), Togo (9), Mali (21), Liberia (6), Sierra Leon (3), Cote D’ivoire (7), Gambia (8), Burkina Faso (22), Guinea (4) Mauritania (2) and Niger (2). No publication was found for Guinea Bissau. After controlling for the inclusion and exclusion criteria, a total of 78 publications across only seven countries were found and analyzed. An overview of the filtration process of articles reviewed is provided in Fig. 2.

### General characteristics of reviewed publications

Publications from only seven West African countries out of the fourteen country articles assessed were included in the review. A total of 77 articles and 2 reports published in the period January, 2009–March 2014 were analyzed. The majority of the articles were from Nigeria (*N* = 39), followed by Ghana (*N* = 23), Burkina Faso (*N* = 8) and Mali (*N* = 4), with only one article from Benin, Sierra Leone and Senegal respectively. The two reports were large studies based on the Demographic Health Surveys (DHS) that had investigated issues of reproductive health needs across low-income countries. The reports contained information from country-specific publications that did not appear during the search. Most studies were conducted in both urban and rural areas. The rest of the studies were carried out in either urban or rural areas only. The majority (*N* = 52) were original studies that collected own data. Few articles (*N* = 26) were based on the DHS data conducted across some countries. Two thirds of the studies had a descriptive aim whiles the rest had predictability aim, or had no clear objectives. Details about the study designs applied in the publications reviewed and the quality of these designs can be found in the Additional file 1: Appendix table section.

Overall, a cross-sectional design was applied in most of the studies, with a variety of investigative modes and population-based surveillance designs. Different instruments to collect qualitative and/or quantitative data were applied. Five articles had secondary data reviews in addition to other techniques. Verbal narratives and use of observation checklist was also detected in three studies. Most studies focused on specific population groups (e.g. rural areas, urban areas or towns/cities). Only two articles reported country-wide studies. A high proportion of the articles had sample sizes less than 500 respondents (*N* = 41) or more than 2000 respondents (*N* = 31). Few studies had 500 to 2000 respondents.

### Specificity of outcome measures in reviewed publications

Table 1 illustrates the outcome measures for three types of reproductive health services reported: antenatal care, contraceptive services and obstetric care. Three themes of outcome measures were distinguished: clinical, economic/geographical and patient-reported outcomes. Clinical based outcomes of antenatal care varied and included quality of service, satisfied demand, out-of-pocket payments, antenatal visits per woman and level of service utilization.

**Table 1:** Cross tabulations of outcome measures and reproductive health need at three levels in reviewed publications

Category of outcome measures	Reproductive health needs at three levels for married and non-married women		
	Antenatal care	Contraceptive use	Obstetric care
Clinical outcomes	1. Number of Antenatal care during pregnancy	1. Service constraints	Critical obstetric danger signs knowledge
	2. Place of delivery(Home/facility)	2. Demand satisfied	Preparations by women prior to delivery and facility type
	3. Supervised or Non-Supervised delivery	3. Provider Intimidation	3. Facility quality and resources
	4. Use of Traditional birth attendant for delivery	4. limited contraceptive choice	4. Obstetric maternal outcomes
	5. Mother survival	5. Nurses withholding information	5. Obstetric utilization/complication
	Newborn survival	6. Method choices at facilities	6. Quality of obstetric care
	Level of utilization and quality of service	7. Unintended pregnancies	
	8. Language of service provision	8. Unmet contraceptive seeking demand	
Economic and geographical outcomes	1. Service utilization for postnatal services	1. Unmet need for contraception	1. Economic access to obstetric care
	2. Economic access to service	2. Service constraints	2. Risk of intrapartum and antepartum still birth
	3. Out-of pockets payments	3. Economic access	3. Place of delivery
		4. Use of modern contraceptives	4. Delay in seeking care
		5. Community level/ecological zone	5. Delay in reaching a health facility
			6. Delay in been provided with appropriate care
Patient-reported outcomes	1. Service constraints	1. Current use	Demand satisfied
	2. Demand satisfied	2. Ever use	Quality of service delivery
	3. Quality of service delivery	3. Never use	Awareness of danger signs
	4. Decision making choices for maternal care	4. Intention for future use	Emergency planning steps awareness by women
	5. Safe delivery	5. Unmet need for contraception	Reasons for seeking abortion and post abortion services
		6. Contraceptive prevalence rates	Unsafe abortions
		7. Proportion of demand satisfied	
		8. Knowledge of contraception use	
		9. Current and Ever use of FP	
		10. Demand satisfied	
		11. Quality of service delivery	

Contraceptive utilization outcomes at the clinical level included the existence of widespread contraceptive shortages coupled with limited choices for contraceptive use by potential users. Unintended pregnancies were widely observed with service constraints and provider attitudes resulting from scolding and intimidation of the clients by providers. At the clinical level, obstetric care was assessed on critical danger signs observed from pregnancy to delivery and preparations by women prior to delivery. Provision of basic emergency comprehensive obstetric care (BEmOC) at the facility, user satisfaction, obstetric and abortion outcomes and knowledge of the health care operatives on standard emergency obstetric care (EmOC) were other reported outcomes. Obstetric maternal outcomes were assessed on maternal complications and live births, as well as on supervised and non-supervised deliveries. Economic/geographical outcome measures included cost associated with access to services, use of services, delays in seeking care, delays in reaching a health facility, delays in receiving appropriate care and place of delivery.

Patient-reported measures in studies on antenatal care assessed women’s satisfaction with service use, service quality, decision making for services used and place of service utilization.

Regarding contraceptive services, unmet need for contraception were measured based on current and ever use of contraception, contraceptive prevalence rates, intention for future use and women’s knowledge of contraception use and benefits. Patient-reported measures related to obstetric care assessed unsafe abortions occurrence, reasons for seeking abortion, post abortion care, complication readiness, emergency planning steps, and awareness of danger signs prior to delivery.

### The extent of unmet need outcomes reviewed in publications

Table 2 summarizes three critical outcome measures reported in the publications reviewed. These outcome measures were mostly assessed and reflect only contraceptive use across clinical, economic and patient levels. Antenatal and obstetric care were not extensively highlighted in the reviewed publications and thus, not included here. As evidenced in Table 2, unmet need for contraceptive use remains high across all countries. Ghana has the highest level of this type of unmet need across West Africa and Nigeria has the lowest rate. Contraceptive prevalence rates are highest in Ghana and lowest in Benin. Modern contraceptive prevalence rate reflects current acceptance and use of modern methods of contraceptive for all age groups. Hence, low rates depict low use and acceptance to modern contraceptive use may translate to low use in the future. Adolescent’s birth rates, though declining, still remain high and stagnant among most adolescent girls in West Africa (see Table 2). Mali and Benin indicated an increase over a five year period (2001–2006). Two countries, Ghana and Burkina Faso showed a remarkably reduction in the rates for various years, however large disparities still exist across all social and economic groups.

**Table 2:** Extent of unmet needs per outcome measures in countries in review

Outcome measure	Country (Year)	Outcome measures	Reference index ^1^
Unmet need for contraception (% of married, fecund or in union women not using contraception; but intends to space or limit births)	Ghana (2008)	35.3 %	1,2,7,11,17,18,21,68,72-78
	Senegal (2005)	31.6 %	30,77,78
	Mali (2006)	31.2 %	32,42,60,69,77,78
	Benin (2006)	29.9 %	67,77,78
	Burkina Faso (2003)	28.8 %	37,51,57,51,66,77,78
	Sierra Leone (2008)	27.6 %	31,77,78
	Nigeria (2008)	20.2 %	77,78
Contraceptive prevalence rate for modern contraceptive (% of women married or in union of reproductive age using modern method of contraception)	Ghana (2008)	16.6 %	1,2,7,11,17,18,21,68,72-78
	Burkina Faso (2006)	13.3 %	30,77,78
	Senegal (2005)	10.0 %	32,42,60,69,77,78
	Nigeria (2008)	8.1 %	67,77,78
	Mali (2006)	6.3 %	37,51,57,51,66,77,78
	Sierra Leone (2008)	6.0 %	31,77,78
	Benin (2006)	5.9 %	77,78
Adolescent birth rates per 1000 girls (number of births per 1000 girls between the ages 15 and 19 years)	Mali (2001–2006)	185 to 188	1,2,7,11,17,18,21,68,72-78
	Sierra Leone (2006)	143	30,77,78
	Burkina Faso (1999–2003)	131 to 119	32,42,60,69,77,78
	Nigeria (2003–2008)	126 to 121	67,77,78
	Benin (2001–2006)	109 to 112	37,51,57,51,66,77,78
	Senegal (1997–2005)	103 to 101	31,77,78
	Ghana (2003–2008)	74 to 66	77,78

Table outcome estimates were extracted from a review of 24 countries in developing countries which included all country context in this review [17]. ^1^A few publications provided data on all countries context

### Specificity on determinants in reviewed publications

The main determinants reviewed in the publications are summarized in Table 3. Socio- cultural determinants were influenced by the perceptions of what constitute marriage and family, thus creating unequal power and gender disparities, affecting mainly women in attaining their needs for reproductive health. Additionally, socio-cultural factors influencing antenatal, contraceptive, and obstetric care utilization were observed to be driven by the desire for a large family size by male spouses, community and individual perspectives of what is socially acceptable and worthwhile for women as well as some religious doctrines surrounding womanhood. Others such as women feeling disempowered owning to their poor quality of life and poor spousal communication, women’s desire to secure their marriages and attract their husband’s love and attention were also observed to stem from socio-cultural beliefs and perspectives.

**Table 3:** Cross tabulations of determinants and reproductive health need at three levels in reviewed publications

Category of determinants	Reproductive health needs at three levels for married and non-married women		
	Antenatal care	Contraceptive use	Obstetric care
Socio-cultural factors	1. Ethnicity and residence of women	1. Women age and parity	1. Cultural acceptability
	2. Cultural viewpoints and beliefs	2. Number of surviving children	2. Social stigma
	3. Family unions of women	3. Spousal communication	3. Socio-demographic factors
	4. Increasing Parity needs	4. Husband refusal to use, socio	
	5. Husband/parental influence	5. cultural and religious beliefs,	
	6. Empowered decision making beliefs	6. desire for large family size	
	7. Religion (ATR)	7. Desire for their husband attention, love and favor	
	8. Social factors	8. Marriage status of women	
Institutional/clinical factors	1. Access by distance	1. Poor health infrastructure	1. Poor health service infrastructure
	2. Economic cost	2. Unfriendly relational attitude of health providers	2. Lack of skilled personnel for obstetric services
	3. Geographic inaccessibility	3. side effects of use	3. Place of delivery
	4. Poor health infrastructure	4. Method choices available for women	4. Physician inadequate to deliver services
	5. Unfriendly attitude of health providers	5. Ineffective leadership in managing and monitoring the demand	5. Optimal organization of obstetric services
	6. Unavailability of health staff at facility		6. Poor and inefficient counselling
	7. Poorly equipped health infrastructure		
	8. Type of facility (private/public/Hop/clinics		
Economic factors	1. Socio-economic status	1. Socioeconomic status of woman	1. Cost effectiveness in accessing services
	2. Household wealth	2. Economic Access for contraceptives	2. Travel cost and distance
	3. Cost of accessing delivery services	3. Residence(rural/urban	3. Catastrophic expenditures
	4. Women value in society		
Knowledge and risk factors	1. Educational Status of women	1. Previous experience	1. Restrictive abortion laws
	2. Knowledge of danger signs	2. Fear of side effects	2. Poor knowledge concerning
	3. High risk patient risk	3. Educational status of woman	3. Lifesaving skills (LSS) for health staff
	4. Risk of associated with utilization of services		

Socio-economic measures such as the socio-economic wealth of a woman or household wealth were found to be key determinants in most publications for access and service use. Even in environments where women owned economic assets and resources, their economic value is seen as reduced since the male spouse socio-culturally is seen to own and control household resources. Socio-economic factor such as money was seen to impact travel cost, cost of service provisions and catastrophic expenditures on household wealth and income.

Poor knowledge concerning lifesaving skills (LSS) by health staff in delivering quality and efficient services was reported in some studies. Low or poor educational status of women and their knowledge of danger signs, knowledge of where to access services and the risk associated with service were also key determinants influencing service utilization in some settings. Conspicuously missing in almost all reviewed publications was the role health provider’s play in influencing the services use through their educational sessions for women at the clinic or community setting as outlined in other studies [25]. Overall, single determinants alone were never observed in publications. Socio-demographic characteristics and institutional factors were the main driving indicators of current and future determinants for service utilization. Thus, the determinants reviewed indicate that, several factors either tend to work towards greater efficient use or deny access to millions of women from attaining their needs to reproductive health.

### Unmet needs and gender constraints identified in publications

Review results show male dominance and authority on reproductive health needs among women in West Africa, which have a strong impact on women’s reproductive health needs [18, 26–32]. As mentioned above, this is driven by socio-cultural customs and beliefs of what constitute marriage, family and reproduction in these settings. These attributes were observed to have created power inequalities and gender disparities, affecting mainly women in attaining their needs for reproductive health [21, 27, 33–35].

The results also point to the huge infrastructure deficits and ill equipped health delivery systems in poor resource settings as a major constraint for women to meet their reproductive health needs [12, 36–40]. Community effects in relation to geography, cultural and religious beliefs are other major obstacles for women’s attainment of their reproductive needs [26, 41]. Certain environments tend to “devalue” women and reinforce strong power relations among men and women. Restrictive abortion laws in some countries also make women resort to adopting clandestine unsafe abortion strategies, which possess greater risk for women to survive later pregnancy and child bearing [38, 42].

## Discussion

### Extent of unmet needs among women in West Africa

The results of this review indicate a high level of unmet reproductive care needs reported by women in West Africa. Overall, contraceptive unmet need levels are a critical outcome measure for women reproductive health across the region. However, the more specific outcome measure of modern methods of contraceptive use is also important if the measure is to remain relevant and precise in capturing gaps for improving contraceptive supplies. We observed in the review that these two indicators can be contradictory. For example, Ghana shows the highest rate for unmet contraceptive needs but also a highest modern contraceptive prevalence rate, compared to the rest of countries in the review. Thus, Ghana has succeeded in increasing women’s awareness of the need of contraceptives but has not achieved universal access to contraceptive services. Similarly, Benin has also a high level of unmet contraceptive need but also a low prevalence rate of modern contraceptive. Thus, efforts to bridge the wide disparities are necessary to make visible progress on meeting women’s health needs. This applies to the rest of the countries as well. Although the use of modern contraceptive is higher in countries such as Burkina Faso and Ghana, rates found in this review are still rather low compared to the rest of the developed world. Further steps need to be taken to improve the reproductive health delivery systems in West Africa.

Indices across West Africa on adolescent birth rates, contraceptive prevalence rate, and unmet need levels reflect poor and lagging policies of fertility, poor accountability in the health sector and the poor socio-economic growth across the region. Although various policies exist across West Africa aimed at improving reproductive health outcomes (as outlined in the introduction section), they are not well implemented and do not assure comprehensive coverage. There is no substantial progress in assuring free and adequate maternity care services. A few advantaged women across these countries may be contributing to national and country context progress on the critical indicators reviewed in this paper.

Thus, current reproductive measures for evaluating reproductive health needs appear broad and do not consider country differences in socio-economic development, political stability, and the health system across countries reviewed. There is a need for context specific outcome measures and determinants for tracking progress in each country. In particular, data are not available for all countries for the same year, and also within a country for several years. There is a need for the development of reliable data by strengthening the health surveillance and information management systems across the health systems in the region.

### Key determinants to women reproductive health in publications

The reviewed publications indicate that social and cultural barriers to the use of reproductive health services are present in all countries although the degree and extent varies by country. Economic access was reported to be a major contributory factor for the use of services by women, depending upon the socio-economic status of women seeking care, household wealth status, and the cost of service provision at the point of use. Even when out-of-pocket payments are not an ultimate determinant of utilization; distances that women have to travel were seen to influence health outcomes such as place of delivery, demand satisfied, and maternal death. The geographic distributions of health staff as a measure further compounds access issues as evidenced in this review. Efforts at bridging access by various governments must start with providing the enabling infrastructure to bridge institutional determinants for intended users.

The effects of women’s educational status and knowledge of risk factors was associated with decisions to adopt or not to adopt services in most reviewed publications for countries such as Nigeria, Ghana, Mali and Sierra Leone. Even in settings where women viewed knowledge as a non-important determinant to meeting their reproductive needs, their inability to read and recollect properly next visit days for services impeded on effective service provision by health providers. Quality and satisfaction of the place of service utilization and the presence of a skilled attendant during service use were observed as determinants and measures used coherently in almost all countries reviewed in this study. These outcomes related to quality and satisfaction, have the tendency to determine the use of care. Improving quality and satisfaction reassures and addresses new users concerns and has the potential to improve women knowledge and use now and in future.

Additionally, our findings also highlight the role of the male spouse in influencing women reproductive behaviors. Poor spousal communication shows the lack of an equal “playing field” for women at the family level in terms of decision making. Traditional family settings in West Africa view the family as a single unit governed by the single head which is the man. Opportunity for dialogue is scarcely available concerning the daily management issues at the family level prescribing men with the social responsibility as breadwinners. In families where women face opposition and obstacles to reproductive service utilization, covert use is sometimes adopted. However, covert use is no option for women using services in settings were privacy is not assured since it can warrant marital problems. This was most evident in strict religious dominated environments and countries such as Nigeria, Benin and Niger whose papers were reviewed.

### Limitations

Although our literature search was systematic and assessed all related studies within the desired scope, it is possible that we missed relevant publications, e.g. publications reported in non-English journals and most recent studies not yet reported. Also, eligible studies were only found for 7 out of the 15 West African countries. No study was found for Guinea Bissau (at least among the English publications). Two countries, Nigeria and Ghana had a relatively high number of studies included in the review compared to the rest of the countries. Despite this limitation on the country coverage and number of publications finally reviewed, the findings of this study remain relevant for the selected West African countries because of the similarities in the social, community and regional context, as well as health systems across the region [43, 44]. In view of this, our conclusions are essential for the improvement of reproductive outcomes in the entire region, including countries where such studies are absent. In addition, the absence or low proportion of studies in some West African countries on the inclusion criteria, as indicated by our review, confirm the need of more policy and research attention to women’s reproductive health needs in the region.

## Conclusion

This paper has focused on analyzing outcome measures and determinants of unmet reproductive care needs in West Africa based on a systematic review of previous studies. The findings from this review show the existence of a broad range of outcome measures and determinants for evaluating reproductive care needs applied across the countries. Evidence shows a high rate of unmet reproductive care needs despite the progress in some countries. This is associated with poor socio-economic indices, deep and varied social norms and beliefs across cultures and traditions that place marriage and a women’s reproductive health needs as the male spouse responsibility at the family unit level. Critical too are the poor institutional arrangements, rising adolescent birth rates, poor economic status and poor knowledge, which further divest women reproductive health seeking behaviors in these societies. The multiple social, economic and environmental factors that affect women meeting their reproductive health needs along the continuum of care, require the involvement of user, provider and policy-makers to bring the desired changes in the countries. In pursuing these ambitions, reforms that improve access (e.g. legislative reforms on restrictive abortion laws) will go a long way to improve unmet need for obstetric care in West Africa. Although outcome measures and determinants identified in the review remain critical progress benchmarks globally, they are rather broad. There is need to incorporate country level context measures in benchmarking progress on reproductive health needs among women to better evaluate each country progress on reproductive health.

## Additional file

Additional file 1:
**General Characteristics of all assessed publications for the review.** (PDF 211 kb)

## Abbreviations

BEmOC: Basic emergency obstetric care
CPR: Contraceptive prevalence rate
DHS: Demographic health surveys
EmOC: Emergency obstetric care
LSS: Life saving skills
PRISMA: Preferred reporting items for systematic reviews and meta-analysis
SSA: Sub-Saharan Africa
