# Supplementary material for: Unmet reproductive health needs among women in some West African countries: a systematic review of outcome measures and determinants
Source: Reprod Health. 2016 Jan 16;13:5. doi: 10.1186/s12978-015-0104-x (PMC4715869; doi:10.1186/s12978-015-0104-x)
Supplement: Additional file 1: — General Characteristics of all assessed publications for the review. (PDF 211 kb) [file 12978_2015_104_MOESM1_ESM.pdf]

## APPENDIX A

### Table 1 General Characteristic of Publication Reviewed (78 publications reviewed) [1]

| Classification Category | Sub-categories | N (%) | Reference index in Appendix A |
|---|---|---|---|
| Type of publication | Journals | 76(59) | 1-76 |
| | Reports, Briefs | 2(2) | 77,78 |
| Year of Publication | 2009-2010 | 26(20) | 1,2,4,6,9,15,20,10,23,25,26,27,28,34,36,37,41,46,52,55,67,68,70,72,73,78 |
| | 2011-2012 | 35(27) | 3,5,10,11,14,16,17,18,22,24,30,31,32,33,35,38,39,40,43,44,47,48,53,54,57,58,59,60,62,64,65,66,69,71,75,77 |
| | 2013-2014 | 17(13) | 7,8,12,13,19,21,29,42,45,49,50,51,56,61,63,74,76 |
| Country of Study | Ghana | 23(18) | 1,2,7,11,13,14,15,17,18,21,22,23,24,29,36,43,50,58,59,68,72,73,76 |
| | Nigeria | 39(30) | 3,4,5,6,8,9,10,12,16,19,20,25,26,27,28,33,39,40,41,44,45,46,47,48,49,52,53,54,55,56,61,62,63,64,65,70,71,74,75 |
| | Burkina Faso | 8(6) | 34,35,37,38,51,57,51,66 |
| | Mali | 4(3) | 32,42,60,69 |
| | Benin | 1(1) | 67 |
| | Sierra Leone | 1(1) | 31 |
| | Senegal | 1(1) | 30 |
| | West Africa | 2(2) | 77,78 |
| Study setting | Rural | 20(16) | 1-3,7,29-31,34,36,37,39,40,42,46,51,55,56,59,65,72 |
| | Urban | 27(21) | 4,8,9,10,12,15,18-23,25,26,33,41,44,47-50,60,63,64,67,70,73 |
| | Urban / rural | 31(24) | 2,5,6,11,13,14,16,17,24,27,28,32,35,38,43,45,53,54,57,58,61,62,44,64,66,68,69,71,74,75,76,77,78 |
| Intent Measures | Outcome measures | 38(30) | 11,13,24,27,28,32,35,38,43,45,53,54,57,58,61,62,1,30,31,36,37,39,59,10,12,18,33,41,44,47,48,48,67,69,71, 74,77,78 |
| | Determinants | 63(49) | 2,5,6,14,16,17,24,27,38,43,45,54,57,58,61,62,1,3,7,29,34,36,37,39,40,42,46,51,52,55,56,4,8,9,12,15, 18,19,20,21,22,23,25,26,41,44,48,49,50,60,63,64,65,66,68,70,72,73,75,76,77,78 |
| Objective of Study | Descriptive | 65(50) | 56,55,59,51,46,42,40,37,36,34,31,30,29,3,1,50,49,48,44,41,26,25,21,19,18,15,12,10,9,8,4,20,63,62,61,58,57, 53,45,43, 38,32,28,27,24,17,16,14,13,11,2,5,64,65,66,68,69,70,71,72,73,74,75,77,78 |
| | Predictive | 9(7) | 52,30,3,60,54,67,76,77,78 |
| | Quantitative | 7(5) | 56,40,60,43,64,77,78 |
| | Evaluatory | 13(10) | 59,55,52,40,39,36,34,7,47,64,76,77,78 |
| | Not clear | 5(4) | 47,33,23,22,35 |

# APPENDIX B

Table 5: SUMMARY CHARACTERISTICS OF ARTICLES AND REPORTS REVIEWD IN STUDY

| Reference/Country of origin/ type of Publication | Objectives of study | Study design/ Data collection /Analytical methods | Reliability Internal validity External validity | Outcomes measures /determinants Identified | Unmet Gaps/Gender constraints identified | Main Findings /conclusions/ policy recommendations |
|---|---|---|---|---|---|---|
| 1<br>Yakong, et al 2010<br>Ghana<br>Peer Reviewed Journal Article | Title: Women experiences of seeking reproductive health care in rural Ghana: Challenges for maternal health service utilization<br><br>Objective:<br><br>Description of rural women perspectives on their experiences in seeking reproductive care from professional nurses | Based on constructivist paradigm (Creswell 2007)<br><br>In-depth Audio taped interviews with individuals and focus group discussions lasting 45-90 minutes.<br><br>Convenience sample of 27 participants (15-49 years)<br><br>Translated, transcribed verbatim, manually coded and analyzed using thematic analysis | *Reliability*<br>clear<br><br>*Internal validity:*<br>clear<br><br>*External validity*:<br>clear<br><br>Limitations:<br>None | *Outcome(s) measures*<br>Women experiences of intimidation and being scolded in meeting their reproductive health<br><br>Women experiences of limited choices to reproductive health needs<br>Women experiences of nurses withholding information from them<br>Women experiences with the lack of privacy<br><br>*Determinant(s)*<br>Unfriendly relational attitude of health providers | *Unmet Gap(s)*<br><br>Limited commodity security<br>Conditions for privacy in the provision of services<br><br>*Gender constraints*<br><br>Dominant discourses and practices in Ghanaian society devalue women and reinforce power relations.<br><br>Women accepted disrespect, intimidation and scolding they received from nurses in meeting their reproductive health care needs in order to obtain the care they need | *Main Findings*<br>1.Healthcare providers relational practice influence women health care seeking behaviors<br><br>2.Women perspectives in seeking health services should be address by structural changes to health clinics and routine practices that create conditions for privacy to address women concerns<br>*Recommendations*<br>1. More emphasis should be placed on raising awareness among nurses about women accounts of care seeking, ethical practices and professional code of conduct.<br>2.Nursing regulatory boards and health |

| | | | | | | |
|---|---|---|---|---|---|---|
| 2<br>Johnson & Madise<br>2011<br>Ghana<br>Peer Reviewed Journal Article | Title:<br>Targeting women at risk of unintended pregnancy in Ghana: should geography matter<br><br>Objective:<br><br>Identify spatial correlation in contraceptive behavior among women | 1998 and 2003 GDHS survey data on 400 enumeration areas and 410 enumeration areas respectively.<br><br>Spatial analysis technique<br><br>Logistic regression modelling | *Reliability*<br>NOT clear<br><br>*Internal validity:*<br>NOT clear<br><br>*External validity*:<br>NOT clear | <u>*Outcome(s)*</u><br><br>Rural versus urban women need<br><br>Household characteristics e.g. spousal communication, education, no of surviving children has an impact on utilization<br><br>Community level/ecological zone of residence affects utilization of services | <u>*Unmet Gap(s)*</u><br>*Contraceptive demand are not fully met*<br><br><u>*Gender constraint(s)*</u><br><br>Study demonstrate significant community effects, which influence the risk of unintended pregnancies for women in the community | ministries must play a major role in ensuring that nurses provide accessible, acceptable and culturally – appropriate care to all users, irrespective of their social conditions<br><br>3.Good role modelling is required for nurses entering practice<br><br>1.Accounting for background demographic and socio economic characteristics, the risk of unintended pregnancies varies significantly between communities within ecological zones of Ghana<br><br>2.Results may reflect increasing disparities in access to contraception in rural areas of the savannah and coastal zones |

| | | | | | | |
|---|---|---|---|---|---|---|
| 3<br>Olugbenga-Bello et al<br>2011<br>Nigeria<br>Peer Reviewed Journal Article | Title: Contraceptive practices among women in rural communities in South Western Nigeria<br><br>Objective: determine CPR among rural women in the study setting | Descriptive cross sectional study<br><br>Questionnaire on 612 women respondents<br><br>SPSS Version 15 | *Reliability*<br>clear<br><br>*Internal validity:*<br>clear<br><br>*External validity*:<br>Clear<br><br>Limitations:<br><br>Not identified during review | *Determinant(s)*<br>Women age, educational status, number of surviving children<br><br>duration since last birth, access and economic factors and frequency of spousal communication<br><br>*Outcome(s)*<br>Current use of contraceptives<br><br>Main reasons for choice of contraceptives<br><br>Reasons for non- use of any method<br><br>Duration of family planning use<br><br>Cost and availability influence method choice.<br>Unmet need for contraception was high with about a quarter of all women studied not on any contraceptive method | Unmet Gaps<br>Not identified in review<br><br>Gender Constraints<br>Cultural and religious beliefs make women non autonomous to seeking their own reproductive health needs | 1. Use of modern contraceptives method was high. Nonuse was attributed to fear of side effects, husband's refusal and the desire for large family size.<br>2.Religious leaders should be targeted and carried along in the campaign for modern contraceptive methods The mass media should do inform in reaching a large populace with the message of contraception. |

| | | | | | | |
|---|---|---|---|---|---|---|
| 4<br>Izugbara<br>2010<br>Nigeria<br>Peer Reviewed<br>Journal Article | Title: Gendered interest and poor spousal contraceptive communication in Islamic Nigeria<br><br>Objective: Barriers to spousal contraceptive communication | Cross sectional<br>In-depth interviews and focus group discussions with women and men<br><br>Muilstage sampling<br>Purposive sampling<br>Qualitative analysis | *Reliability*<br>clear<br><br>*Internal validity:*<br>clear<br><br>*External validity*:<br>clear<br><br>Limitations:<br>Study did not match views of couples. Juxtaposing the views and beliefs of couples on spousal communication would have yielded more critical insights on the study. Data were only based on qualitative views of FGDs and IDIs,NOT backed by participants observations and creating a level of ethnographic slenderness. | <u>*Determinant(s)*</u><br>Husband refusal to use, socio cultural and religious beliefs, desire for large family size and fear of side effects of use<br><u>*Outcome(s)*</u><br><br>High parity was<br><br><u>*Determinant(s)*</u><br>Desire for their husband attention, love and favor influence non use<br><br><br>Large family size was a determinants to marriage security for women<br><br>Spacing or limiting could endanger ones position in marriage | <u>*Unmet Gaps*</u><br><br>Men's desire for large family sizes bares them from accepting<br><br><br><u>*Gender Constraints*</u><br>Religious beliefs among Muslims about large family sizes with resort to women health and reproductive status<br><br>Women are only respected if they have high parity | Married women use their high parity capacities as a micro-tactic for economic survival among male dominated population that is influenced by religion<br><br>The existence of such religious beliefs and systems make it difficult for women to have their desired reproductive needs met<br><br><br>This also creates poor spousal communication since women and men don't negotiate with regards to family planning |

| | | | | | | |
|---|---|---|---|---|---|---|
| 5<br>Adebayo et al 2013<br>Nigeria<br>Peer Reviewed Journal Article | Title:<br>Modelling Geographical variation and determinants of use of modern family planning methods among women of reproductive age in Nigeria<br><br>Objective:<br>Examining the determinants to Family planning use | Survey method National HIV/AIDS and reproductive health survey (NARHS) 2003,2005,2007 dataset of 12,538 respondents<br><br>Women (15-49years) and men 15-64 years Stratified sampling techniques<br><br>Regression modelling used in analysis | *Reliability*<br>clear<br><br>*Internal validity:*<br>clear<br><br>*External validity*:<br>clear<br><br>Limitations:<br><br>Not indicated in publication | *Outcome(s)*<br>Educational status influence use of family planning<br><br>Never married women desire for use increased highly (93%) compared to married women<br><br>*Determinant(s)*<br><br>Marriage status<br><br>Educational status<br><br>socioeconomic status for married women | *Unmet Gaps*<br><br>Not identified in review<br><br>*Gender constraints*<br><br>Not identified in review | 1.Considerable geographical variations were found in the use of family planning in Nigeria(rural/urban, access and non-access to service points)<br>2. Use of FP had positive association with Maritl status and economic status.<br>3.Women respondents never married were two times higher likely not to use modern FP compared with current/former married<br>4. Efforts should be target at behavior maintenance for current users.<br><br>5.Economic empowerment may be a factors for use of FP among Married women but cannot be said of unmarried women |

| | | | | | | |
|---|---|---|---|---|---|---|
| 6<br>Omeje, J. et al<br>2011<br>Nigeria<br>Peer Reviewed Journal Article | Title:<br>Does possession of assets increase women's participation in reproductive decision-making? perceptions of Nigerian women<br><br>Objective:<br>Assess the effects of wealth ( assets) on RH decision making at the HH level | Population based descriptive questionnaire survey. Structured questionnaire to Collect data on 150 married women.<br>Multistage sampling<br>Random sapling<br>Purposive sampling<br><br>SPSS Version 12 used for analysis | *Reliability*<br>clear<br><br>*Internal validity:*<br>clear<br><br>*External validity*:<br>clear<br><br>*Limitations:*<br><br>Not identified in review | <u>*Outcomes(s)*</u><br><br>Women ownership of household assets does not improve her power to bargain for her reproductive needs<br><br>Men "still remain the sole" drivers to when and how to FP use<br><br><u>*Determinant(s)*</u><br><br>*Social norms exerts an influence on use even with other positive factors* | <u>*Unmet gaps/gender constraints*</u><br><br>Women have low bargaining reproductive health rights.<br><br>Gender bias exist in the ownership of assets and thus prescribes as a social norm the responsibility of men as breadwinners and determinants to health and other fertility issues at the household level. This has important implications for women bargaining power in reproductive decision making. | 1.RH decision making among couples is complex and affected by several factors extending beyond asset procession<br><br>2. By controlling financial and household assets, men control reproductive decision making at the household level.<br><u>Recommendations</u><br>1. Family planning Policy makers must mainstream this component to tackle social norms that see women as non-owners of assets.<br>2. Programs may target empowering women (economic, social, political etc.)<br>3.Male participation strategies should be upscale to improve acceptance and approval by men for women Service providers at antenatal and postnatal clinics must provide more method choices to improve acceptability and use for previous users |

| | | | | | | |
|---|---|---|---|---|---|---|
| 7<br>Eliason et al<br>2013<br>Ghana<br>Peer Reviewed Journal Article | Title: Factors influencing the intention of women in rural Ghana to adopt postpartum family planning<br><br>Objective:<br>To examine influencing factors to postpartum family planning use | Survey technique among antenatal attendees to clinics in 4 health facilities in Mfantseman district<br>1900 women targeted | *Reliability*<br>clear<br><br>*Internal validity:*<br>clear<br><br>*External validity*:<br>clear<br><br>*Limitations*<br>Study did not follow upon pregnant women intention for PPFP to see if they were met | *Outcome measure(s)*<br>Knowledge of implant and importance of exclusive breastfeeding are associated with intention to adopt PPFP<br>Previous user before birth were more likely to adopt PPFP services<br>Prior users of IUD were unlikely to adopt PPFP<br>*Determinant(s)*<br>Past experiences of use(IUD as a method was likely to reduce adoption of PPFP for previous users )<br><br>Formal education.<br>Acceptability by husbands.<br>Method choices | *Unmet Gaps*<br>*Not identified*<br><br><br>*Gender constraints*<br>High proportion of women (82%) indicated they will require the permission of their partners to adopt a method | Women empowerment within the context of her household and daily life and relations with her partner can play a powerful role in women likelihood to use a modern contraceptive method. |

| | | | | | | |
|---|---|---|---|---|---|---|
| 8<br>Carroon, Meghan et al<br>2014<br>Nigeria<br>Peer Reviewed | Title:<br>The role of Gender Empowerment on Reproductive Health Outcomes in Urban Nigeria<br><br>Objectives:<br>To examine gender empowerment and its role in reproductive health in Nigeria | Baseline household survey data from the measurement/learning and evaluation project for the Nigerian Urban Reproductive Health Initiative | *Reliability*<br>clear<br><br>*Internal validity:*<br>clear<br><br>*External validity*:<br>clear<br><br>*<u>Limitations</u>*<br><br>Empowerment measurement based on married women only<br>Cross sectional and did not intend to draw on causality measures between gender and reproductive health use | *<u>Outcomes</u>*<br><br>Women empowerment<br><br>*<u>Determinants</u>*<br>Economic access (money of their own)<br><br>Empowered women tend to use family planning more<br><br>Geographical location affects how money and economic empowerment will be | *<u>Unmet Gaps</u>*<br>Gender as a construct limits access and use in Muslim dominated settings<br><br>*<u>Gender constraints</u>*<br>Muslim dominated settings in the study did not have considerations for gender. Use (current and ever) use were all low in these areas | 1.Disparities existed between various geographical locations of study (Abuja, Northern cities and Southern cities)<br><br><u>Recommendations</u><br><br>Need to identify other influencing factors of contraceptive apart from gender effects which did not matter in the Abuja results compared to the Northern and Southern results. (geography has a role to influence kind of limiting factors that may be present) |

| | | | | | | |
|---|---|---|---|---|---|---|
| 9<br>Utoo et al<br>2010<br>Nigeria<br>Peer Reviewed<br>Journal Article | Title:<br>Knowledge ,attitudes and practice of family planning methods among women attending antenatal clinic in Jos,North-Central Nigeria<br><br>Objective:<br>To examine KAP for antenatal care in Nigeria | Cross sectional survey involving 420 women attending antenatal clinics<br><br>Structured interview questionnaire was used<br>Analysis done Not clear | *Reliability*<br>clear<br><br>*Internal validity:*<br>clear<br><br>*External validity*:<br>clear<br><br>*Limitations:*<br><br>Not identified in review | <u>*Outcome measure(s)*</u><br>Most women received their family planning information at the clinic (60%).<br>A high proportion of women (75%) agreed that both spouses should take collective decisions on their RH needs.<br>Intention for future use was high (68%)<br><br><u>*Determinant(s )*</u><br>Desire for more children accounts for non-use in the future<br>Educational status<br>Adequate knowledge<br>Acceptability of FP methods by family | <u>*Unmet Gaps*</u><br><br>Not identified in review<br><br><u>*Gender constraints*</u><br><br>Not identified in review | 1.Despite high educational status, knowledge and acceptability by known and new users, CPR is still low<br><br>2.Targeted interventions on how to translate these knowledge and acceptability levels to attain high RH outcomes |

| | | | | | | |
|---|---|---|---|---|---|---|
| 10<br>Olamijulo et al<br>2012<br>Nigeria<br>Peer Reviewed<br>Journal Article | Title: knowledge and practice of contraception among pregnant women attending antenatal clinic in Lagos University Teaching Hospital. | Cross sectional survey with pregnant women<br><br>Questionnaire to 151 pregnant women at clinics | *Reliability*<br>clear<br><br>*Internal validity:*<br>clear<br><br>*External validity*:<br>clear<br><br>Limitations:<br>Not identified in the review | *Outcomes*<br>No plans for use after delivery<br>*Determinants*<br>No association was established between PPFP use and age, religion, educational attainment | Unmet Gaps<br>Not determined in study<br><br><br>Gender Constraints<br>Not detected in the study | 1.Knowledge about Contraceptives and where to obtain them is not a challenge to most women in the study. There was no association between age, education parity and desire for PPFP use<br>2.Information dissemination about benefits of child spacing and provision of incentives for easy uptake of post-partum contraception would help to correct this imbalances |
| 11<br>Amoako J et al<br>2012<br>Ghana<br>Peer review<br>Journal Article | Title/objective:<br>Estimating unmet need for contraception by district within Ghana: An application of small area estimation techniques | 2003 GHDS and 2000 household survey / population and housing census in Ghana | *eliability*<br>clear<br><br>*Internal validity:*<br>clear<br><br>*External validity*:<br>clear<br><br>Limitations:<br>Not identified in review | Outcome measures<br>Levels of unmet needs across and within district<br>*Determinants*<br>Lack of availability of contraceptives. Inadequate absorptive capacity. Poor resource mobilization. Ineffective leadership in managing and monitoring the demand and supply of contraceptive methods | *Unmet Gaps*<br><br>Unmet needs exist<br><br>*Gender constraints*<br><br>Not identified in review | 1. There is considerable geographical variation in contraceptive use, unmet need and satisfaction of demand for contraception is high.<br>2.SAE Techniques could be potential useful at the district level ti inform about use of contraceptives rather than regional figures provided in DHS |

| | | | | | | |
|---|---|---|---|---|---|---|
| | | | | | | 3.Variations exist by district in use and unmet levels<br>CPR varies from 4.1% to 41.7% whiles use varies from 4% to 34.8% |
| 12<br>EO Asekun-Olarinmoye et al 2013<br>Nigeria<br>Peer reviewed Journal Article | Title/Objective:<br>Barriers to use of modern contraceptives among women in an inner city area of Osogbo metropolis, Osun State, Nigeria | Descriptive cross sectional study<br>Self- administered Questionnaire on 359 women (WIFA)<br>Multi-stage sampling design<br><br>SPSS Ver. 17 | *Reliability*<br>clear<br><br>*Internal validity:*<br>clear<br><br>*External validity*:<br>clear<br><br>Limitations:<br>None | <u>*Outcome measures*</u><br>desire for more children is an indicator of desire for future fertility among women<br>perceived benefits brings influence use<br>Perceived side effects influence use too<br>Ever use greater than Current use shows discontinuation over time<br><u>*Determinants*</u><br>Current/Ever use, fertility preferences, no of children living with, side effects<br>Marital status<br>Awareness of FP service center provisions<br>High education | Unmet Gaps<br><br>Not identified in review<br><br><br><br>Gender Constraints<br><br>Not identified in review | 1.Several socioeconomic and health facility related barriers exist and affect women use of FP<br>Knowledge and awareness are not enough in the presence of social barriers to improve use of FP<br><br>2.Policy interventions should target and institute community based behavioral change communication programs that will tackle these sociocultural barriers |

| | | | | | | |
|---|---|---|---|---|---|---|
| 13<br>Asamoah et al 2013<br>Ghana<br>Peer Reviewed Journal Article | Title/Objective: Inequality in fertility rate and modern contraceptive use among Ghanaian women from 1988-2008 | Constructed a database using the women questionnaire from the GDHS 1988,1993,2003,2008 datasets<br><br>Regression based total attributable fraction (TAF)<br>Relative and slope indices of Inequality (RII and SII) | *Reliability*<br>clear<br><br>*Internal validity:*<br>clear<br><br>*External validity*:<br>clear<br><br>Limitations<br><br>Not detected in the review | <u>*Outcomes measures*</u><br><br>Non-use or ever use of contraceptives<br>Fertility rates<br><br><u>*Determinants*</u><br><br>Education<br>Maternal age<br>Residence<br>Marital status<br>Income level | Unmet Gaps<br>Not identified in the review<br><br><br>Gender constraints<br><br>Not identified in the review | 1.Increasing trend in fertility rate related to education, income and residence from 1988 to 2008 despite a decrease in trend in contraceptive use over the period<br>2.Significant education and income related inequalities exist that affects contraceptive use Women with basic education are affected most in inequality rather than residence and income levels<br>3.Targeted programmes and policies an modern contraceptive use should be introduced in Ghana that will specifically focus on the contraceptive needs of women with up to basic education Fertility education should be incorporated in basic school curriculum<br>Gaps identified<br>More research into the causes of the unfortunate discrepancy is urgently needed |

| | | | | | | |
|---|---|---|---|---|---|---|
| 14<br>Crissman et al<br>2012<br>Ghana<br>Peer Review<br>Journal Article | Title/Objective:<br>Women sexual empowerment and contraceptive use in Ghana | 2008 GDHS data on socio-demographics and other health indices. | *Reliability*<br>clear<br><br>*Internal validity:*<br>clear<br><br>*External validity*:<br>clear<br><br>Limitations:<br>Not identified from review | <u>*Outcome measures*</u><br>Unmarried women current use<br>Ever use<br><br><u>*Determinants*</u><br>Religion(Muslim have decrease use)<br>Education<br>Increasing wealth | <u>*Unmet Gaps*</u><br>Unmet needs exist in areas where gender relations disempower women from seeking their RH needs<br><br><u>*Gender Constraints*</u><br>Gender disparities in sexual empowerment for women still hinder | 1.Unmarried women were less to use Contraceptives in study.<br>2.Gender disparities exist in women meeting their RH need for contraception use<br>Economically disadvantaged women needs in gender disparities must be addressed |
| 15<br>Adanu et al<br>2009<br>Ghana<br>Peer Reviewed<br>Journal Article | Title/Objective:<br>Contraceptive use by women in Accra, Ghana: Results from the 2003 Accra women Health Survey | Cross sectional study<br>Self-administered questionnaires for 2199 women<br><br>STATA 8.2 | *Reliability*<br>clear<br><br>*Internal validity:*<br>clear<br><br>*External validity*:<br>clear<br><br>Limitations:<br>Not identified in review | <u>*Outcome measures*</u><br><br>Use of contraceptives influenced by educational status<br><br><u>*Determinants*</u><br><br>Female education | Unmet Gaps<br><br>Not identified in review<br><br>Gender constraints<br><br>Not identified in review | 1. Regular use of health facilities does not guarantee that women will take up Contraceptive use now or later.<br>2. Educational status was the most significant predictor to contraceptive use.<br>Female education must be incorporated into educational system to empower women for future desire for more fertility options |

| | | | | | | |
|---|---|---|---|---|---|---|
| 16<br>AKPA,O.M. et al<br>2012<br>Nigeria<br>Peer Review<br>Journal Article | Modeling the determinants of fertility among women of Childbearing age in Nigeria: Analysis using generalized linear modeling approach | 2008 NDHS on 20,974 women data were extracted and analyzed using descriptive statistics and Poisson regression | *Reliability*<br>clear<br><br>*Internal validity:*<br>clear<br><br>*External validity*:<br>clear<br><br>*Limitation:*<br>Not identified during study | *Outcome measures*<br><br>Measures of fertility<br><br>*Determinants*<br>Income levels<br>Residence(rural/urban)<br>Less educated | Unmet Gaps<br>Not identified in review<br><br>Gender constraints<br><br>Not identified in review | 1.High Fertility levels were found among rural women, less educated and poorer women. This is attributed to the desire for large family size by most rural women.<br>2.Desire could also be precipitated by the husbands request even though women may want to postpone or stop child bearing at all. Deep sociocultural norms and values that eulogize large family size must be addressed if women are to meet their fertility desires. |
| 17<br>Kodzi et al<br>2012<br>Ghana<br>Peer Review<br>Journal Article | Title/Objective:<br>To have or not to have another child: life cycle, health and cost considerations of Ghanaian women | Individual level prospective study using longitudinal data (1998-2003)<br><br>Modeling for predictors | *Reliability*<br>clear<br><br>*Internal validity:*<br>clear<br><br>*External validity*:<br>clear | Outcome measures<br>Child bearing<br><br>Determinants<br>Previous experience<br>Partner influence<br>Material conditions<br>Religion | Unmet Gaps<br>Not identified in review<br><br>Gender Constraints<br>Spousal discussion is low or absent in most cases for women | 1.Interventions targeting to improve contraceptive use must target women with low levels of education<br><br>2.Interventions must target the negative attitudes towards FP use |

| | | | | | | |
|---|---|---|---|---|---|---|
| 18<br>R.M. Adanu<br>et al<br>2012<br>Ghana<br>Peer review<br>Journal Article | Title:<br>Sexual and reproductive health in Accra.Ghana<br><br>Objective:<br>To descript sexual and reproductive health among women in Accra and explore the burden of sexual and reproductive health | Cross sectional study<br>Data from WHSA-II (N=2814)<br>Supplemented by household survey (n=400),focus group discussions (n=22) and in-depth interviews (n=20) among women | *Reliability*<br>clear<br><br>*Internal validity:*<br>clear<br><br>*External validity*:<br>clear<br><br>Limitations:<br><br>Not identified in survey | <u>*Outcome measures*</u><br><br>Ever use<br>Accuracy of information and benefits they will drive<br>Many method choices drive use and change anytime you want to<br><br><u>*Determinants*</u><br><br>Not living with partner<br>Wealth<br>Age | Unmet Gaps<br><br>Wide disparity between knowledge and use<br><br>Gender constraints<br><br>Not identified in review | Despite increasing economic development and declining fertility, modern contraceptives methods use remains low<br><br>It is important to improve upon commodity security to sustain and remove lapses of users due to shortages |
| 19<br>Chibuike.O.Chigbu et al<br>2013<br>Nigeria<br>Peer review<br>Journal Article | Title/Objective:<br>Denial of women right to contraception in Southeastern Nigeria | Cross sectional survey with 1204 women registered for prenatal care services<br>Questionnaire was used | *Reliability*<br>clear<br><br>*Internal validity:*<br>clear<br><br>*External validity*:<br>clear | <u>Outcome measures</u><br>Access and use of contraception<br><br><u>Determinants</u><br><br>Husband approval | <u>*Unmet Gaps*</u><br>43.4% had unplanned pregnancy due to lack of support from husbands to use contraception<br><br><u>*Gender constraints*</u><br>Women education or awareness level of their reproduce rights could help in them meeting their desired contraceptive use | 1.Right to contraception was not known by nearly half of participants<br>Approve of joint decision making on family fertility issues (84.1%)<br>2. Majority of previous users were used because their husbands opted for them to (61.9%)<br>3.Majority of women in the study will like to take part in family planning decision making. |

| | | | | | | |
|---|---|---|---|---|---|---|
| | | | | | | However, most of them are denied these rights leading to appreciable number of unwanted pregnancies. Although female education may increase their awareness of their reproductive rights, it may not affect their ability to exercise these rights.<br>Education of men on women reproductive rights is important |
| 20<br>Igwegbe AO et al<br>2010<br>Nigeria<br>Peer Review Journal Article | Title:<br>Knowledge and practices of family planning among antenatal care attendees at Nnewi, South east Nigeria<br><br>Objective:<br>To determine knowledge and practice of family planning among women in south east Nigeria | Cross sectional survey. pre-tested interviewer questionnaire was used to collect data from respondents | Reliability:<br>clear<br>Internal Validity:<br>Clear<br><br>External validity:<br>Clear<br>Limitations:<br>Not identified in study | Outcome measures<br>Ever use of Family planning<br>Knowledge of Family planning<br>Method choices<br><br><br>Determinants<br><br>Benefits of family planning | Unmet Gaps<br><br>Not identified in review<br><br>Gender Constraints<br><br>Not identified in review | 1.76.5% has ever used modern method of contraception. 95.5% knew about contraception.<br>2.birth spacing(72.9%), limiting(40.6%) and its importance in reduction in maternal mortality (1.7%) were some reported benefits why they use family planning |

| | | | | | | |
|---|---|---|---|---|---|---|
| 21<br>Joseph Kofi<br>2013<br>Ghana<br>Peer Review<br>Journal Article | Title:<br>Modern contraceptive use among women in the Asuogyman district of Ghana: Is reliability more important than health concerns<br><br>Objective:<br>The study examines the socio-demographic determinants of modern contraceptive use among women in Ghana | Cross sectional survey | Reliability:<br>clear<br>Internal Validity:<br>Clear<br><br>External validity:<br>Clear<br>Limitations:<br>Not identified in study | Outcome measures<br><br>Knowledge of contraception<br><br>Current use<br><br>Barriers to use<br><br>Determinants<br>Education<br>Place of residence<br>Work status | Unmet Gaps<br><br>Not identified in review<br><br><br><br>Gender Constraints<br><br>Not identified in review | 1.97% of survey respondents knew of at least one modern method of contraception, only 16% of them were using contraceptives.<br>2.Statistical test show that education, place of residence and work status influence modern contraceptive use among women<br>3. Fear of side effects, desire for more children and partner disapproval are main barriers that prevent use |

| | | | | | | |
|---|---|---|---|---|---|---|
| 22<br>Biney AA<br>2011<br>Ghana<br>Peer Review<br>Journal Article | Title:<br>Exploring contraceptive knowledge and use among women experiencing induced abortion in the Greater Accra Region ,Ghana<br><br>Objectives:<br>To examine contraceptive use and knowledge among abortion seekers | Qualitative research methodology using semi-structured interviewer questionnaire | Reliability:<br>clear<br>Internal Validity:<br>Clear<br><br>External validity:<br>Clear<br>Limitations:<br>Not identified in study | Outcome measures<br><br>Knowledge of contraception<br><br>Contraceptive failure<br><br><br>Determinants<br><br>Contraceptive failure<br>Side effects after use | Unmet Gaps<br>Not identified I review<br><br><br><br><br>Gender Constraints<br><br>Not identified in review | 1.Women tended not to have knowledge about contraceptive methods prior to the abortion<br>2.fear of side effects and other health reasons prevented others with knowledge from using them<br>Recommendations<br>Peer and reproductive health education must be reinforced in communities |
| 23<br>Adenu RM<br>2009<br>Ghana<br>Peer Review<br>Journal Article | Title:<br>Contraceptive use by women in Accra; results from the 2003 women health survey<br><br>Objectives:<br>To determine the predictors to modern contraception use in Ghana | Household survey of women in the 2003 women health survey in Accra.<br>Data was collected with questionnaire from 2199 women | Reliability:<br>clear<br>Internal Validity:<br>Clear<br><br>External validity:<br>Clear<br><br>Limitations:<br>Not identified in study | Outcome measures<br><br>Significant Predictor(s )<br>Ever use<br><br>Determinants<br>Education | Unmet Gaps<br>Not identified in review<br><br><br><br>Gender constraints<br>Not identified in review | 1.Education was the most significant predictor of use<br>2.women without education had a 48% reduction in the odds of having ever used contraception and 66% reduction in the odds of currently using contraception<br>Recommendations<br>Female education needs to be emphasized and promoted. |

| | | | | | | |
|---|---|---|---|---|---|---|
| 24<br>Abdul-Rahaman et al<br>2011<br>Ghana<br>Peer Review Journal Article | Title:<br><br>Trends in contraceptive use among female adolescents in Ghana<br><br>Objectives<br><br>To examine trends in contraceptives use in Ghana | 2003 and 2008 GDHS data | Reliability:<br>clear<br>Internal Validity:<br>Clear<br><br>External validity:<br>Clear<br>Limitations:<br>Not identified in study | Outcome measure<br><br>Current use<br><br>Modern versus traditional options<br><br>Intention for future use<br><br>Determinants<br>Barriers<br>Access to commodities<br>Cost<br>Misconceptions about health effects | Unmet Gaps<br>Identified unmet need for contraception due to increasing barriers.<br><br>Gender Constraints<br>Not identified in review | 1.increase in use from 23.7% in 2003 to 35.1% in 2008<br>2. There was a shift from modern methods to traditional contraceptive methods<br>3.There was a slight decline 7%(4.4%points) in the number of non-users who intended to use contraceptives in the future. |
| 25<br>Nte AR et al<br>2009<br>Nigeria<br>Peer review Journal Article | Title:<br><br>Male involvement in family planning: women perception<br><br>Objective:<br>To assess knowledge of mothers of under five children of male involvement in family planning. | Cross sectional descriptive survey Involved a questionnaire interview of mothers | Reliability:<br>clear<br>Internal Validity:<br>Clear<br><br>External validity:<br>Clear<br>Limitations:<br>Not identified in study | Outcome measures<br><br>Contraceptive prevalence rates<br>Women perceptions on male involvement for family planning<br>Discontinuation due to male influence<br><br>Determinants<br>Husband approval, access | Unmet Gaps<br><br>Not identified in review<br><br>Gender Constraints<br><br>Not identified in review | 1.CPR s was 5.6% and 85.6% knew at least one method for males<br>2.52.7% currently using will discontinue if their husbands object to the use<br>3.22.1% of women interviewed felt male involvement could impact on their acceptance rate of family planning services<br>4.Male spouses despite being older, educated |

| | | | | | | |
|---|---|---|---|---|---|---|
| | | | | | | and better paid jobs and in charge of their reproductive health at home, did not contribute to the knowledge of the women and their practices of family planning |
| 26<br>Okereke CI<br>Nigeria<br>Peer review<br>Journal Article | Title:<br>Unmet reproductive health needs and health seeking behavior of adolescents in Owerri, Nigeria<br><br>Objective:<br>Examined unmet reproductive health needs and health seeking behavior of adolescents in Nigeria. | Cross sectional survey with the use of questionnaire | Reliability:<br>clear<br>Internal Validity:<br>Clear<br><br>External validity:<br>Clear<br>Limitations:<br>Not identified in study | Outcome measures<br><br>Unintended pregnancies<br><br>Recurrent pregnancies<br><br>Abortion<br><br>Primary contact with unmet needs<br><br>Determinants<br>Access<br>cost | Unmet Gaps<br><br>Not identified in review<br><br>Gender Constraints<br><br>Not identified in review | 1.30.2% of respondents had had unintended pregnancies.<br>2.73.3% have had recurrent pregnancies whiles 19.6% have had an abortion carried out before<br>3.Medicine operators was the first primary contact for health care |

| 27<br>Reference/Country of work/ type of Publication | Title/Objectives of study | Study design/ Data collection /Analytical methods | Reliability Internal validity External validity | Outcomes measures /determinants Identified | Unmet Gaps/Gender constraints identified | Main Findings /conclusions/ policy recommendations |
|---|---|---|---|---|---|---|
| Babalola et al 2009<br>Nigeria<br>Peer reviewed Journal Article | Title:<br>Determinants of use of maternal health services in Nigeria-Looking beyond individual and household factors.<br><br>Objective(s):<br>Examination of maternal service utilization in Nigeria with a focus on individual, HH, community and state level factors | Interviewer-administered nationally representative survey (2005 National HIV/AIDS and Reproductive Health Survey).<br><br>Household questionnaire was administered to provide quantitative data on reproductive health interventions in Nigeria.<br><br>Multi-level Analytical models used measures at individual, household, community and state levels. | *Reliability*<br>clear<br><br>*Internal validity:*<br>clear<br><br>*External validity*:<br>clear<br><br>*Limitations*<br><br>NARHS study was based on self-reports of respondents, and provide no validation of obtained information with an objective source such as hospital or clinic records. | *Outcome measures*<br><br>Use of maternal health services<br><br>Supervised delivery<br><br>Use of postnatal care services<br><br>*Determinants*<br>Age of mother<br>TBAs<br>Education<br>Ethnicity and residence<br>Socio-economic status | Unmet Gaps<br><br>Not identified in study<br><br>Gender Constraints<br><br>Not identified in study | 1. The study revealed several predictors to maternal health service use under three areas; use of maternal care (60.3%) at least once during last pregnancy, skilled delivery (43.4%) whiles 41.2% received postnatal care.<br>2. Utilization is higher than skilled delivery.<br>3. Important predictors stem from institutional to geographic, economic and socio-cultural barriers that affect use.<br>Recommendations<br>1.Support for multi-level approaches; individual, community and household level programs will help |

| | | | | | | |
|---|---|---|---|---|---|---|
| 28<br>Egbewale et al<br>2009<br>Nigeria<br>Peer Reviewed<br>Journal Article | Title:<br>Demographic profile of mothers and their utilization of maternal health-care services in Osun State, Nigeria<br><br>Objectives:<br>To examine the current level of utilization of maternal health care in some rural and peri-urban communities in the study setting | Cross sectional descriptive study<br><br>House to house survey using semi-structured questionnaire to interview adult women<br><br>Analysis not identified | *Reliability*<br>clear<br><br>*Internal validity:*<br>clear<br><br>*External validity*:<br>clear<br><br>*Limitations reported:*<br><br>Not identified | *Outcome measures*<br><br>Place of delivery<br>Supervised delivery<br><br>*Determinants*<br><br>Socio-economic status<br>Educational level<br>Cultural viewpoints /beliefs | Unmet Gaps<br><br>Poor access to health centers and distances to access health facilities is still a challenge for most rural women.<br><br>Gender Constraints<br>Not identified | 1.Majority of women delivered in health care facilities which were supervised by skilled attendants.<br><br>2. Two key predictors were seen to influence uptake of services. Educational level and economic status of women.<br><br>Recommendation(s)<br><br>Continuous and sustained community education and mobilization is essential so that women and their families learn about the need for special care during pregnancy and childbirth under trained health personnel |

| | | | | | | |
|---|---|---|---|---|---|---|
| 29<br>Crissman, H.P. et al 2013<br>Ghana<br>Peer reviewed Journal Article | Title:<br>Shifting norms: pregnant women's perspectives on skilled birth attendance and facility-based delivery in rural Ghana.<br><br>Objectives:<br><br>To examine pregnant women perspectives of skilled and facility based delivery in study setting | Cross sectional descriptive study.<br><br>85 pregnant women attending antenatal clinics<br><br>Analyzed using ground theory methodology (Interview transcripts) | *Reliability*<br>clear<br><br>*Internal validity:*<br>clear<br><br>*External validity*:<br>clear<br><br>*Limitations identified:*<br>Not identified | *Outcome measures*<br><br>Skilled delivery<br>Facility delivery<br><br>*Determinants*<br><br>Cost associated with delivery despite waived facility fees<br><br>Treatment by service providers<br><br>Community support for pregnant mothers increasing<br><br>Access by distance resulting in economic cost | Unmet Gaps<br><br>Poor access to health centers and distances to access health facilities is still a challenge for most rural women.<br><br>Gender constraints<br><br>Not identified | There is increasing evidence from this research of increasing support of community for Health care facility (HCF) delivery in Ghana.<br><br>Recommendation(s)<br><br>Barriers that still remain in the fullest utilization of these services should be addressed by policy makers and implementers to promote safer motherhood at using services |

| | | | | | | |
|---|---|---|---|---|---|---|
| 30<br>Faye, Adama et al 2011<br>Senegal<br>Peer Reviewed Journal Article | Title:<br>Home birth in women who have given birth at least once in a health facility: Contributory factors in a developing country<br><br>Objective:<br><br>Study link between patient satisfaction about received services in health facilities and the choice of future delivery place of women who had deliveries at least once in a facility | Cross sectional descriptive study with 373 women who gave birth in last 12 months before survey<br><br>Questionnaire was used<br><br>Logistic regression Analysis was used | *Reliability*<br>clear<br><br>*Internal validity:*<br>clear<br><br>*External validity*:<br>clear<br><br>*Limitations*<br><br>Prevalence of home births may be underestimated because only surviving women were interviewed. Also ,satisfaction is a subjective concept that can vary from person to person that may not reflect the intrinsic quality of care | *Outcome measures*<br><br>Place of delivery<br><br>Demand satisfied individuals<br><br>Sex of skilled attendant<br><br>Quality of service delivery<br><br>*Determinants*<br><br>Family unions of women<br><br>Transportation to health delivery centers<br><br>Distance lived from facility<br><br>No of already antennal visits to facility | *Unmet Gaps*<br><br>Quality remains pervasively compromised in service provision<br><br>*Gender constraints*<br><br>Not identified | 1. Home births were common among women in polygamous families and parity greater than 3.<br>2. Place of delivery was influenced by person conducting the deliveries and quality of delivery to be expected.<br>3. Satisfaction was related to the place where the patient previous birth took place.<br><br>Recommendations<br>Emphasis should be placed on training healthcare providers to improve the quality of service provided to patients in health facilities. Psychological support and reception of patients should be incorporated into training for health caregivers |

| | | | | | | |
|---|---|---|---|---|---|---|
| 31<br>Oyerinde et al<br>2012<br>Sierra Leone<br>Peer Reviewed<br>Journal Articles | Title:<br>A Qualitative Evaluation of the choice of Traditional Birth Attendants for Maternity Care in 2008 Sierra Leone: Implications for Universal Skilled Attendance at Delivery<br><br>Objective(s):<br>Identify why women use services provided by TBAs as compared to health facilities and to suggest strategies to improve utilization of health facilities | Cross sectional study<br><br>Qualitative data with focus groups in urban and rural areas in july 2008 for men(64) and women(96)<br><br>Qualitative analysis based on content analysis.<br><br>Majors themes were transcribed into English transcripts that were manually coded and tabulated | *Reliability*<br>clear<br><br>*Internal validity:*<br>clear<br><br>*External validity*:<br>clear<br><br>*Limitations*<br><br>Limited Quality assurance on filed workers due to prevailing insecurities situation at that time in study setting | *Outcome measures*<br><br>Use of TBAs for delivery<br><br>*Determinants*<br><br>Geographic inaccessibility<br><br>Trust for TBAs over health staff<br>Prohibitive Cost of services<br><br>Absent staff from health centers<br><br>Poor health infrastructure<br><br>Perceptions that facilities were poorly equipped to provide them services | Unmet Gaps<br>Not identified<br><br>Gender Constraints<br>Not identified | 1. The determinants in this study all contributed to influencing women to opt for TBA delivery instead of health facility.<br>2. Until the quality of service at health facility is seen as better than services provided by TBAs, the cost in time, dignity and money associated with using health facilities will outweigh the benefits and serve as barriers to the utilization of health facilities.<br>**Recommendations**<br><br>Comprehensive and respectful care for women, free from financial considerations, appropriate health infrastructure and facilities should be bridged if services at health facilities are to see a scale up at the community level. |

| | | | | | | |
|---|---|---|---|---|---|---|
| 32<br>Dogba et al<br>2011<br>Mali<br>Peer Reviewed Journal Article | Title:<br>Mother and newborn survival according to point of entry and type of human resources in a maternal referral system in Kayes (Mali)<br><br>Objectives:<br>To assess women with obstetric need use of referral system on maternal and child survival<br>Effects of configuration of healthcare team at the CHCs on joint mother –newborn survival. | Cross sectional study with 7,214 women using referral systems from 2006-2009<br><br>Software analysis employed using SPSS Version 15 and STATA ver. 9<br><br>Bivariate Probit equation and Probit bivariate regression used | *Reliability*<br>clear<br><br>*Internal validity:*<br>clear<br><br>*External validity*:<br>clear<br><br>*Limitations*<br><br>Study did not assess travel distance to access care. This can be a proxy determinant in accessing services.<br><br>Study did not explore individual factors eg education, economic status among others to service delivery and access.<br><br>Survival rates estimates used are an overestimation of survival rates since the hospital cannot even | *Outcome measures*<br>*Entry level*<br>Mother survival<br>Newborn survival<br>Quality of care<br>CHC Team<br><br>*Determinants*<br><br>Size of health care team<br>type(regional/community clinic)<br><br>treatment (current/prior caesarean history, transfusion)<br>age of mother<br><br>presence of physician in health facility | Unmet Gaps<br><br>Not identified in study<br><br>Gender constraints<br><br>Not identified in study | 1.Probalibity of mother survival was associated with new born survival (rho=0.45,Chi-square =96.47,p=0.0000)<br>2. Women who came from far(more than 50 km ) away from the facility ,only those who went to the Regional Hosp (RH) had a joint mother-newborn survival (11.90%) higher than those going to CHCs p<0.001)<br>3.Going to the RH from a distance of 5km or less was associated with the best probability of joint survival(14.51% higher than the reference group ,p<0.001)<br>**Recommendations**<br>1.Although Community health centers (CHCs) do not have the technological platform that would allow them to provide specialized emergency obstetric care, the presence of qualified personnel at this peripheral level is a determine factors for mother-child survival ,especially for women who |

| | | | | | | |
|---|---|---|---|---|---|---|
| | | | guarantee an estimation of 100% point estimates of mortality rates(usually given up to 80%) | | | live in remote areas and access to the health system is in a poor health condition.<br><br>2. Upgrading the skill of the CHC personnel may bring useful benefits to improve maternal and newborn care in these remote areas<br>3. Ensuring that, facilities remain close about 3km or less to community reach can go a long way to improve maternal-newborn survival conditions in poor resource areas. |

| | | | | | | |
|---|---|---|---|---|---|---|
| 33<br>Doctor, H.V. et al 2013<br>Nigeria<br>Peer Reviewed Journal Article | Title: Awareness of critical Danger Signs of Pregnancy and Delivery, Preparations for Delivery, and Utilization of Skilled Birth Attendants in Nigeria<br><br>Objective(s): Document associations between knowledge of critical obstetric danger signs, preparation for birth and delivery by an SBA.<br><br>Document how maternal socio-economic status affects maternal birth delivery services | Population based quantitative household survey<br><br>Household Questionnaire incorporated many closed ended questions identical to those of the Nigeria DHS 2008<br><br>Analysis done with STATA Version 11 | *Reliability*<br>clear<br><br>*Internal validity:*<br>clear<br><br>*External validity*:<br>clear<br><br>*Limitations*<br><br>Recall bias was possible, present with any other retrospective survey. Efforts were made to correct these before, during and after data collection | *Outcome measures*<br><br>Critical obstetric danger signs observed<br><br>Preparations for women prior to delivery<br><br>Delivery by skilled attendant<br><br>Socioeconomic status influence on maternal delivery<br><br>*Determinants*<br><br>Parity<br>Place of residence<br>Socio-economic status | Unmet Gaps<br><br>Not identified in study<br><br>Gender Constraints<br><br>Not identified in study | 1.Findings in study conclude that few women sought ANC (24.7%), 63.5% made at least one preparation for child birth. Only 14% knew at least one critical pregnancy sign compared with 80% of women who knew of at least one non-critical danger sign.<br>2 Existing knowledge did not include some of the serious danger signs. Those who had ANC and knowledge of critical signs were more likely to be attended by skilled attendant and had safe births at the facility.<br>**Recommendations**<br>Tackling the challenges require multi-level and multi-focal approaches. barriers, educations for women at the community and local level of importance of ANC and challenges with the poor health system should be improved (Timely and quality EmOC, poor transport system |

| | | | | | | |
|---|---|---|---|---|---|---|
| 34<br>De Allegri, M. et al 2011<br>Burkina Faso<br>Peer Reviewed Journal Article | Title:<br>Determinants of utilization of maternal care services after the reduction of user fees: A case study from rural Burkina Faso<br><br>Objective(s):<br>To determine the utilization for antennal care (ANC) and skilled attendance at birth after a substantial reduction in user fees. | Cross sectional survey<br><br>Data was collected using a household survey<br><br>Three stage cluster sampling<br><br>Analysis done with STATA IC 11 | *Reliability*<br>clear<br><br>*Internal validity:*<br>clear<br><br>*External validity*:<br>clear<br><br>*Limitations*<br><br>Indicators on the quality of maternal health services available were lacking in this study. Previous research has shown that quality of care may shape and influence women decision to use ANC and skilled delivery. | Outcome measures<br><br>ANC Utilization(at least 3 ANCs)<br>Skilled delivery<br><br>*Determinants*<br><br>Living distance to facility<br>Religion(ATR)<br>Women literacy<br>Household wealth<br>Ethnicity<br>Previous ANC attendance | Unmet Gaps<br><br>Not identified<br><br>Gender Constraints<br><br>Not identified | 1. Multivariate odds indicate living 5km to facility has positive association with service use. ATR, ethnicity and higher HH wealth were negatively also associated with ANC use.<br>2. Delivery service use was affected by ethnicity; distance to facility (5km) and haven attended at least 3 ANCs visits during pregnancy.<br>3.These findings indicate the reduction of user<br>Free or low cost to emergency transport and the wider availability of skilled attendance at birth closer to women residency is important.<br>Further research<br>Why after user fee reduction, use of ANC and facility delivery still differs across ethnic and religious groups. |

| | | | | | | |
|---|---|---|---|---|---|---|
| 35<br>Allegri et al<br>2012<br>Burkina Faso<br>Peer Review<br>Journal Article | Title:<br>The impact of targeted subsidies for facility-based delivery on access to care and the equity- Evidence from a population-based study in rural Burkina Faso<br><br>Objectives:<br>To assess the impact of the new financing policy on subsidy of facility based deliveries on maternal service utilization in Burkina Faso | Cross sectional survey<br><br>Before and after study design<br><br>data from 2006-2010 among 1050 Households<br><br>Three stage multi cluster sampling techniques<br><br>Logistic regression analysis(SPSS) | *Reliability*<br>clear<br><br>*Internal validity:*<br>clear<br><br>*External validity*:<br>clear<br><br>*Limitations*<br>Recall bais (amounts spent on past health needs)<br><br>The sample strategy used may have resulted in over estimation of utilization levels. | Outcome measures<br><br>No of deliveries<br>Level of utilization<br><br><br><br>*Determinants*<br><br>User fee abolition | Unmet Gaps<br><br>Not identified in study<br><br><br><br><br>Gender Constraints<br><br>Not identified in study | 1. Percentage of women delivering in a health facility increased significantly from 49 to 84 % (p<0.001) between 2006 and 2010.<br>2. Increase in utilization detected before and after policy implementation favored women with high income.<br>3. Inequities in access remained unchanged after policy introduction<br>3.30% increase in utilization was when reduction (80%) was introduced. 80% subsidy resulted in an increase in utilization not very different from neighboring countries that had achieved a free user conditions for women.<br>Recommendations<br>1. Total elimination of user fees, leading to a free use of services by all pregnant women must be pursued. Whiles this in not guaranteed to tackle use challenges, policy efforts at improving institutional and provider barriers must be |

| | | | | | | |
|---|---|---|---|---|---|---|
| | | | | | | tackled from a multi-sectorial approach.<br>**2.**Further research interest to understand how this free or reduction in service utilization affects quality of service provided<br><br>1.High rate of facility |

| | | | | | | |
|---|---|---|---|---|---|---|
| 36<br>Crissman et al<br>2011<br>Ghana<br>Peer Reviewed Journal Article | Tile:<br>Intentions to deliver in a health facility and healthcare facility based delivery rates among women in Akwatia, Ghana<br><br>Objective:<br>To investigate the beliefs, delivery intentions and subsequent delivery locations among women receiving prenatal care in Ghana | Cross sectional<br><br>Qualitative data from pregnant women attending antenatal.<br><br>Convenience sampling was used<br><br>Interviews were transcribed verbatim into English and thematic themes were developed. | *Reliability*<br>clear<br><br>*Internal validity:*<br>clear<br><br>*External validity*:<br>clear<br><br>*Limitations*<br><br>small sample size, single sight, potential for selection bias and short follow up period | Outcome measures<br><br>Facility delivery<br><br>Determinants<br><br>Religion<br>Family union type<br>Parity | Unmet Gaps<br>Not identified<br><br>Gender Constraints<br><br>Not identified | delivery was observed in the study (86.1%).36 confirmed delivery at facility and 5 unconfirmed locations<br><br>2. All women interviewed said they wanted to give birth in health facility.<br><br>Recommendations<br>Further research is needed to ascertain why and the prevailing conditions that lead to this. High levels of socioeconomic status and higher education however prevented issues as religion, family type and parity rates to affect facility facility delivery |

| | | | | | | |
|---|---|---|---|---|---|---|
| 37<br>Brazier et al 2009<br>Burkina Faso<br>Peer Reviewed Journal Article | Title:<br><br>Improving poor women access to maternity care: Findings from a primary care intervention in Burkina Faso<br><br>Objectives:<br>Assessing maternal health intervention to promote use of maternal care, before during and after delivery services among women | Baseline and end line population based surveys in two districts<br><br>Operations Research Project<br><br>Two stage sampling design was used<br><br>STATA Version 9 And SPSS version 15 for analysis | *Reliability* clear<br><br>*Internal validity:* clear<br><br>*External validity*: clear<br><br>*<u>Limitations</u>*<br>It is difficult to attribute causality to the interventions of the project to improve maternal care services. Difficulty ascertaining what "skilled care" means. staff with essential competencies and supportive enabling environments(adequate supplies, equipment and infrastructure, functional emergency referral system and supportive policies and regulations | <u>Outcome measures</u><br><br>Place of delivery<br><br><u>Determinants</u><br><br>Wealth of woman | Unmet Gap<br>Not Identified<br><br>Gender Constraints<br><br>Not Identified | 1. The intervention helped in an increased in utilization whiles almost eliminating use differentials between wealth quintiles.<br>2. The study showed that the strong effect of wealth on a women use of delivery care was attenuated (diminished), and at endline, the poorest women in the intervention district were equally likely to deliver at a health facility as women in the richest wealth quintile irrespective of age and educational levels.<br>3. This study offer an additional equity rationale for investing in and upgrading primary health care facilities at the local level for the poor.<br>4. They poor do not use hospitals, but will use health services when access and quality are improved at primary care facilities-even in the absence of recommended interventions as free maternal delivery services, health insurance |

| | | | | | | |
|---|---|---|---|---|---|---|
| | | | Third challenge is the impossibility to attribute mortality reduction to programme interventions since maternal mortality is not a good indicator for judging intervention effectiveness. | | | ,cash transfers, loans that lessen the financial burden of maternity care-seeking or create incentives for care seeking<br><br>Recommendations<br>MDG five can be accelerated with more donor funding in poor resource settings to improve quality and access for the rural poor to improve maternal health. |

| | | | | | | |
|---|---|---|---|---|---|---|
| 38<br>Ameur et al<br>2012<br>Burkina Faso<br>Peer Reviewed<br>Journal Article | Title:<br>User fee exemptions and excessive household spending for normal delivery in Burkina Faso: the need for careful implementation<br><br>Objectives: compare total cost of giving birth in health centers offering partial exemptions versus those with full exemptions to assess the impact on additional out-of-pocket –fees for maternal service use | Case control study<br><br>Natural experiment<br>Survey questions were based in similar study in 2006 by IMMPACT and 2010 by VR and AB in Ouargaye ( Ridde et al 2012<br><br>Household asset and Household characteristics were used to measure traditional poverty status( Lindelow M. 2006)<br><br>Random samples of 50 women per 12 health centers were selected with a total of 870 women | *Reliability*<br>clear<br><br>*Internal validity:*<br>clear<br><br>*External validity*:<br>clear<br><br>*Limitations*<br>Potential for recall bias<br>Lack of data in the two studied districts Before the HELP intervention in 2008 | Outcome measures<br><br>Out-of pockets payments<br><br>Determinants<br><br>Cost of accessing delivery services | Unmet Gaps<br>Not identified<br><br>Gender Constraints<br>Not identified | 1. Elimination of fees from facility based delivery benefits the poorest households. The existence of excessive spending related to direct cost of giving birth is of concern, making it urgent for the government to remove all direct fees for obstetric and neonatal care.<br><br>2. However, the policy of completely abolishing user fees is sufficient; the implementation process must have a thorough monitoring system to reduce implementation gaps |

| | | | | | | |
|---|---|---|---|---|---|---|
| 39<br>Moore et al<br>2011<br>Nigeria<br>Peer Reviewed<br>Journal Article | Title:<br>Utilization of health care services by pregnant mothers during delivery: a community based study in Nigeria<br><br>Objectives:<br>Determine the level of utilization of health services by pregnant women during delivery in river state of Nigeria | Cross sectional design<br><br>Questionnaire based survey with 112 mothers aged 15-49 years. | *Reliability*<br>clear<br><br>*Internal validity:*<br>clear<br><br>*External validity*:<br>clear<br><br>*Limitation:*<br>None identified | Outcome measures<br><br>Utilization levels of services<br>Place of delivery<br><br>Determinants<br><br>Long distance to facility<br><br>Unavailability of transport<br><br>Unfriendly attitude of health providers<br><br>Unavailability of health staff at facility<br><br>Lack of urgency at facility<br>Previous uneventful delivery at health facility | Unmet Gaps<br><br>Not identified<br><br>Gender Constraints | 1. Utilization of delivery service is still poor. 2.64(57.1%) used a health facility whiles 48(42.9%) did not.<br>Recommendations<br><br>Concerted efforts should be made at community and government levels to improve utilization through tackling some of the reported barriers to their use to improve delivery outcomes. |

| | | | | | | |
|---|---|---|---|---|---|---|
| 40<br>Moyer et al<br>2012<br>Ghana<br>Peer Reviewed Journal Article | Title:<br><br>Clean delivery practices in rural northern Ghana: a qualitative study of community and provider knowledge, attitudes and beliefs<br><br>Objectives:<br>Study to explore hand washing/use of gloves ,delivery on a clean surface, sterile cord cutting, appropriate cord tying, proper code care following delivery and infant bathing and cleanliness to promote safe delivery outcomes | Cross sectional design<br><br>253 respondents were interview<br><br>In-depth interviews and FGDs were audiotaped, transcribed and analyzed using NVivo. | *Reliability*<br>clear<br><br>*Internal validity:*<br>clear<br><br>*External validity*:<br>clear<br><br>*Limitations:*<br><br>Interviews were conducted by student graduates.it is possible there was a disconnect between community members and students particularly because of language difference. Design did not also include actual infection rates | <u>Outcome measures</u><br><br>Personal hygiene of mother and surrounding to promote clean birth<br><br><u>Determinants</u><br><br>Hand washing/gloves use<br>Delivery surface<br>Bathing/cleanliness<br>Cord dressing<br>Cord cutting and tying | Unmet Gaps<br><br>Not identified in study<br><br>Gender constraints<br><br>Not identified in study | 1.Fair understanding of the need for clean birth is good.<br>2. Overarching themes emerging from the study included the increasing use of facility based delivery, the disconnect between healthcare providers and the community, and the critical role grandmothers play in ensuring clean delivery practices.<br>3. Educating health care providers of harmful traditional practices so they can specifically addressed, strengthening facilities and incorporating influential community members such as grandmothers to ensure success. |

| | | | | | | |
|---|---|---|---|---|---|---|
| 41<br>Olayemi et al 2009<br>Nigeria<br>Peer Reviewed Journal Article | Title:<br>Male participation in pregnancy and delivery in Nigeria: A survey of antenatal attendees<br><br>Objectives:<br>Assess the level of participation of men in pregnancy and birth, the attitudes of the woman and likely targets for improved delivery. | Descriptive Cross sectional study<br><br>462 women attending antenatal<br><br>STATA was used to analyze | *Reliability*<br>clear<br><br>*Internal validity:*<br>clear<br><br>*External validity*:<br>clear<br><br>*Limitations*<br><br>None | <u>Outcome measures</u><br><br>Male participation<br><br><u>Determinants</u><br><br>Age of man<br>Educational status | Unmet Gaps<br><br>Not identified<br><br><br>Gender Constraints<br>Not identified | 1. Male attendance and participation in the study is high which is good according to the survey findings for encouraging and improving birth outcomes.<br><br>2. Husband attendance at ANC services was not high compared to husbands helping at delivery and husbands carrying out other household chores.<br><br><u>Recommendations</u><br><br>Need to emphasize counselling prior to service provision<br><br>Care-givers and hospital administrators need to refrain from hostile policies towards family delivery. |

| | | | | | | |
|---|---|---|---|---|---|---|
| 42<br>White D et al<br>2013<br>Mali<br>Peer Reviewed Journal Article | Title:<br>The influence of intrafamilial power on maternal health care in Mali: perspectives of women, men and mothers-in-laws<br><br>*<u>Objectives:</u>*<br><br>How does intrafamilial power dynamics influence maternal health practices (men/women and mother in-laws) | Cross sectional<br><br>317 Household in two districts<br><br>Multivariable analysis | *Reliability*<br>clear<br><br>*Internal validity:*<br>clear<br><br>*External validity*:<br>clear<br><br>*Limitations*<br><br>*None* | <u>Outcome measures</u><br><br>Quality of service<br><br><u>Determinants</u><br><br>Mother in laws opinions<br><br>Self-efficacy<br><br>Women value in society | <u>Unmet Gaps</u><br>Not identified<br><br><br><u>Gender Constraints</u><br>Gender norms that disadvantage women in seeking care is prevalent in this setting | 1. Women self-efficacy and the quality of services they receive at facility were associated with health seeking practices of mothers.<br><br>2. Preference and opinions of mother in laws were reflected in the maternal behaviors of daughter in laws.<br>3. Interventions focusing on women or couples may be insufficient to advance women RH in patriarchal societies.<br><u>Recommendations</u><br><br>Further research and interventions are needed to address gender norms, and consider influence of other family members on women RH needs |

| | | | | | | |
|---|---|---|---|---|---|---|
| 43<br>Smith ME et al<br>2012<br>Ghana<br>Peer Review<br>Journal Article | Title:<br>Why some women deliver in health institutions and others do not: a cross sectional study of married women in Ghana, 2008.<br><br>Objectives:<br>Explore relationship of some socio-economic variables with institutional delivery | Cross sectional<br><br>Qualitative approach design | *Reliability*<br>clear<br><br>*Internal validity:*<br>clear<br><br>*External validity*:<br>clear<br><br>*Limitations*<br><br>Not identified | <u>Outcome measures</u><br><br>Place of delivery<br><br><u>Determinants</u><br><br>Socio-economic variables<br>Wealth<br>Educational status of women and husbands<br>Women status | Unmet Gaps<br><br>Not identified<br><br><br><br>Gender Constraints<br><br>Not identified | 1.The findings indicate that, a woman's status does not act independently to affect her choice of place of delivery but these are channeled through some socio-economic variables.<br><br>2. Wealth and educational status of women and their partners influenced positively the choice of delivery. Expansion of economic opportunities for women , as well as female education must be encouraged together with male education. |

| | | | | | | |
|---|---|---|---|---|---|---|
| 44<br>Sule ST<br>2012<br>Nigeria<br>Peer Reviewed Journal Article | Title:<br>Utilization of delivery services in Zaria, northern Nigeria: Factors affecting choice of place delivery<br><br>Objectives:<br>Determine effect of companionship during labour and delivery, and the preferred delivery position, on the choice of place of delivery among women in Zaria, with a view to providing more acceptable services | Cross sectional survey<br><br>315 women attending antenatal clinics at primary clinics | *Reliability*<br>clear<br><br>*Internal validity:*<br>clear<br><br>*External validity*:<br>clear<br><br>*Limitations:*<br>*None* | <u>Outcome measures</u><br><br>Choice of delivery place<br><br><u>Determinants</u><br><br>Too expensive to deliver at facility<br><br>Unfriendly providers | Unmet Gaps<br>Not identified<br><br>Gender Constraints<br>Not identified | 1. Women value Social values and support and freedom to decide the position to adopt during labour and delivery.<br>2. Socioeconomic barriers to access and institutional challenges that makes women perceive the clinic setting as unfavorable for delivery must be addressed.<br><br><u>Recommendations</u><br>Curricula for training health staff should incorporate various domains social support and provider patient engagement modalities to enable providers improve the relationships with clients. |

| | | | | | | |
|---|---|---|---|---|---|---|
| 45<br>Ononkpono DN et al<br>2013<br>Nigeria<br>Peer Reviewed Journal Article | Title:<br>Contextual determinants of maternal health care service utilization in Nigeria.<br><br>Objectives:<br>Examine the relation of community factors moderated the association between individual characteristics and antenatal care visits | Data from 2008 NDHS among 16,005 women aged 15-49years who had last delivery last 5 years before survey.<br><br>Multi-level models applied in analysis | *Reliability*<br>clear<br><br>*Internal validity:*<br>clear<br><br>*External validity*:<br>clear<br><br>*Limitation*<br>Not identified in study | <u>Outcome measures</u><br><br>Hospital delivery<br><br><u>Determinants</u><br><br>No of antenatal visits during pregnancy<br><br>Residence (high poverty communities) | Unmet Gaps<br>Not identified<br><br>Gender Constraints<br>Not identified | 1. Living in communities with a high proportion of educated women was not significantly associated to antenatal care visits.<br>2. Community factors acted as moderators of the association between educational attainment and antenatal care attendance.<br><br><u>Recommendations</u><br>Improvement in antenatal care utilization may therefore be enhanced by targeting poverty reduction programs and increasing health facility delivery in disadvantaged communities |

| | | | | | | |
|---|---|---|---|---|---|---|
| 46<br>Oladapo OT et al<br>2009<br>Nigeria<br>Peer Reviewed Journal Article | Title:<br>Do sociodemographic characteristics of pregnant women determine their perception of antenatal care quality?<br><br>Objectives:<br>To explore sociodemographic determinants of perceived quality of antenatal care at the Primary Care level. | Survey of 452 randomly selected pregnant women accessing antenatal care at primary care facilities.<br><br>Bivariate and multivariate logistic regression analysis carried out | *Reliability*<br>clear<br><br>*Internal validity:*<br>clear<br><br>*External validity*:<br>clear<br><br>*Limitations*<br>Not identified by review | Outcome measures<br><br>Quality of antenatal care<br><br>Determinants<br><br>Islamic Religion<br><br>Increasing Parity<br><br>No of living children<br><br>Gainful employment of clients | Unmet Gaps<br><br>Not identified<br><br>Gender Constraints<br>Not identified | 1. The study suggests that sociodemographic characteristics of women have limited impact on their perception of ANC quality.<br>2. Identified predictors may serve as the criteria for selecting women that require intensive health center-specific antenatal interventions aimed at improving perceived quality and thus sustained utilization of antenatal care services in these primary health care facilities. |

| | | | | | | |
|---|---|---|---|---|---|---|
| 47<br>Okafor II et al<br>2011<br>Nigeria<br>Peer Reviewed<br>Journal Article | Title:<br>Impact of free maternal and child Healthcare programme on maternal and neonatal healthcare outcome in Enugu State of Nigeria<br><br>Objectives:<br><br>This study examined the uptake of obstetric services following introduction of free maternal and child health care in Enugu state university in Southeast Nigeria and its impact on maternal and neonatal healthcare outcome. | Retrospective comparative study<br><br>Data on women and their neonates were collected from the Medical Records department of the hospital | *Reliability*<br>clear<br><br>*Internal validity:*<br>clear<br><br>*External validity*:<br>clear<br><br>*Limitations:*<br><br>Not identified by review | <u>Outcome measures</u><br><br>Delivery rates<br><br>Place of delivery<br><br><u>Determinants</u><br><br>Cost (free service factor) | Unmet Gaps<br><br>Not identified<br><br>Gender Constraint<br>Not identified | 1. Free maternal and child health care (FMCHC) caused tremendous increases in the uptake of antenatal booking (202.2%) and hospital delivery (1051.8%).<br><br>2.FMCHC should be adopted by all countries to help improve maternal and neonatal health(MDGs goal 4 and 5) |

| | | | | | | |
|---|---|---|---|---|---|---|
| 48<br>Nwosu BO et al<br>2012<br>Nigeria<br>Peer Reviewed<br>Journal Article | Title:<br>Proximate determinants of antenatal care utilization among women in southeastern Nigeria<br><br>Objectives:<br>To determine the proximate factors that affect utilization of antenatal care among market women in Nnewi, Southeastern Nigeria | Cross sectional study with 398 studied market women<br><br>Questionnaires were instruments for data collection<br><br>Analysis method not clear | *Reliability*<br>clear<br><br>*Internal validity:*<br>clear<br><br>*External validity*:<br>clear<br><br>*Limitations:*<br><br>Not identified in review | <u>Outcome measures</u><br><br>ANC attendance<br>Choice of place (private and public)<br><br><u>Determinants</u><br>Cost<br>Friendly staff from the private hospitals<br>Staff at private available all the time<br>Proximity to facility | Unmet Gaps<br><br>Not Identified<br><br>Gender Constraints<br><br>Not identified | 1.ANC attendance was high as expected in the study (97.2%).most however went after their third trimester (64.5%) and from the private specialist hospital (37.4%), private general practice hospital (34.7%) and government (17.8%)<br><br>2.There is need for research and policy to address the negative attitudes of staff providers towards clients in government hospitals to improve patient relationship and attendance to government hospitals |

| | | | | | | |
|---|---|---|---|---|---|---|
| 49<br>Saidu R. et al<br>2013<br>Nigeria<br>Peer Reviewed Journal Article | Title:<br>An assessment of essential maternal health services in Kwara State, Nigeria<br><br>Objectives:<br><br>To evaluate the levels of emergency obstetric care signal functions in health facilities in a developing in a developing setting with high mortality and morbidity<br><br>To determine if differences exist between private and public health facilities in terms of availability of signal functions | Survey of health facilities in 6 out of the 16 local government areas<br><br>Interviewer-administered facility assessment questionnaire adapted<br><br>Survey questionnaire adopted from WHO/UNFPA/UNICEF | *Reliability*<br>clear<br><br>*Internal validity:*<br>clear<br><br>*External validity*:<br>clear<br><br>*Limitations:*<br>Not identified by review | <u>Outcome measures</u><br>Facility type(private/public)<br><br>Availability of obstetric services<br><br>Utilization levels<br><br>Inequity in geographic access<br><br><u>Determinants</u><br><br>Risk of associated with utilization of services | Unmet Gaps<br><br>Not identified in study<br><br>Gender Constraints<br><br>Not identified in study | 1. Availability of EOC was more among the private sector and this was statistically significant. The study showed that, all stakeholders involved in reducing maternal mortality have a big challenge in the areas of availability, inequity in geographical distribution of EOC and the poor utilization of these EOC services by women. |

| | | | | | | |
|---|---|---|---|---|---|---|
| 50<br>Udofia EA et al<br>2013<br>Ghana<br>Peer Reviewed Journal Article | Title:<br>Birth and emergency planning: a cross sectional survey of postnatal women at Korlu Bu Teaching Hospital, Accra Ghana.<br><br>Objectives:<br>To determine birth and emergency planning steps, awareness of obstetric danger signs, reported maternal and newborn complications and birth outcomes based on length of hospital stay | Facility based postnatal survey among 483 childbearing women | *Reliability*<br>clear<br><br>*Internal validity:*<br>clear<br><br>*External validity*:<br>clear<br><br>*Limitations:*<br><br>Not identified during review | <u>Outcome measures</u><br><br>Awareness of danger signs<br><br>Reported complications of delivery<br><br>Emergency planning steps<br><br><u>Determinants</u><br><br>Knowledge of danger signs | Unmet Gaps<br>Not identified<br><br>Gender Constraints<br>Not identified | 1. Supervised antenatal care and delivery were nearly universal. Overall, 62% had a birth plan, 74% had adequate knowledge of danger signs, and whiles 64% and 37% reported maternal and newborn complications respectively.<br>2. Accomplishments by a birth companion and saving money were considered the most useful planning steps prior to delivery.<br>3. Knowledge of danger signs was associated with with birth and emergency planning, and the birth and emergency planning was associated with reported birth outcome. Birth and emergency planning as a critical component of antenatal care can influence birth outcomes and should be extended to all pregnant women. |

| | | | | | | |
|---|---|---|---|---|---|---|
| 51<br>Some et al 2013<br>Burkina Faso<br>Peer Reviewed Journal article | Title:<br>How decision for the seeking maternal care is made-a qualitative study in two rural medical districts of Burkina Faso<br><br>Objectives:<br><br>This research examines how decisions for maternal care are made in two rural communities in Burkina Faso | Qualitative study<br>FGDs, IDIs<br>30 women were interviewed.<br>Analysis was by QSR Nvivo 2.0 | *Reliability*<br>clear<br><br>*Internal validity:*<br>clear<br><br>*External validity*:<br>clear<br><br>*Limitations*<br>FGDs was difficult to do separately for women without the presence of their husbands. Some with men presence could have influenced certain pattern of response creating some level of bias | <u>Outcome measures</u><br>Decision making choices for maternal care<br><br><u>Determinants</u><br>Fees exemption<br>Cost sharing for services<br>Women empowerment | <u>Unmet Gaps</u><br><br>Not identified in review<br><br><u>Gender Constraints</u><br><br>Women empowerment is a challenge confronting utilization of services by women | 1. Decision making for obstetric care in the family follows the logic of the family management.<br><br>2. Husbands, brothers in laws and parents in laws make the decisions about whether to use a health facility for antenatal care or for delivery. In general, decision makers are those interviewed, because of their social role and status.<br><u>Recommendations</u><br>To increase use of health facilities in the district, there is the need for the empowerment of women could be helpful |

| | | | | | | |
|---|---|---|---|---|---|---|
| 52<br>Onayade, A.A. et al 2010<br>Nigeria<br>Peer Reviewed Journal Article | Title:<br><br>Birth preparedness and emergency readiness plans of antenatal clinic attendees in Ile-ife, Nigeria<br><br>Objectives:<br><br>To assess adequacy of BP/CR plans of antenatal clinic in maternal emergency. | Cross sectional study<br><br>Questionnaire used for data collection<br><br>400 women attending antenatal clinics were recruited for the study<br><br>SPSS version 11 for analysis | *Reliability*<br>clear<br><br>*Internal validity:*<br>clear<br><br>*External validity*:<br>clear<br><br><br>*Limitations*<br>*Not identified in* | <u>Outcome measures</u><br><br>Complication readiness<br><br>Birth preparedness<br><br>Savings for pregnancy<br><br>Place of delivery<br><br><u>Determinants</u><br><br>Knowledge of women on BP/CR | Unmet Gaps<br><br>None<br><br>Gender Constraints<br><br>None | 1.61% of the pregnant women studied made adequate preparations for delivery whiles 4.8% were ready for emergency /complications.<br><br><u>Recommendations</u><br>It is recommended that greater emphasis be given to emergency/complication readiness during antenatal care sessions. |

| | | | | | | |
|---|---|---|---|---|---|---|
| 53<br>Erim et al<br>2012<br>Nigeria<br>Peer<br>Reviewed<br>Journal<br>Article | Title:<br>Assessing health and economic outcomes of interventions to reduce pregnancy-related mortality in Nigeria<br><br>Objectives:<br>Benefits and cost effectiveness of individual and integrated packages of interventions to prevent pregnancy-related deaths | Country and region specific data were synthesized using a computer-based model<br><br>Adopted a previous validated maternal mortality model to Nigeria<br><br>Models –global maternal health policy model | *Reliability*<br>clear<br><br>*Internal validity:*<br>clear<br><br>*External validity*:<br>clear<br><br>*Limitations:*<br><br>Not identified during review | <u>Outcomes measures</u><br><br>Clinical events (pregnancies, live births, maternal complications)<br><br><u>Determinants</u><br><br>Increasing Family planning access and use | Unmet Gaps<br><br>Not identified during review<br><br>Gender Constraints<br><br>Not identified during review | 1. Increasing family planning was the most effective individual intervention to reduce pregnancy related mortality was cost saving and cost effective and prevented nearly 1 in 5 abortions related deaths.<br>2. Integrated and stepwise strategies such as skilled deliveries, facility births, access to antenatal/postpartum care, improved recognition of referral need, transport and availability quality of EmOC, family planning and safe abortion could help prevent 4 out of 5 pregnancy related deaths.<br><u>Recommendations</u><br><br>Family planning should be promoted at all levels to improve pregnancy related outcomes |

| | | | | | | |
|---|---|---|---|---|---|---|
| 54<br>Fawole AO et al<br>2012<br>Nigeria<br>Peer Reviewed<br>Journal Article | Title:<br>Predictors to maternal mortality in institutional deliveries in Nigeria<br><br>Objectives:<br><br>To determine risk factors for maternal mortality in institutional births in Nigeria | Analytical cross sectional design<br><br>Stratified multi stage sampling used<br><br>SPSS Ver. 15 for analysis | *Reliability*<br>clear<br><br>*Internal validity:*<br>clear<br><br>*External validity*:<br>clear<br><br>*Limitations*<br><br>Not ide stated in Publication | <u>Outcome measures</u><br><br>Maternal mortality<br><br><u>Determinants</u><br><br>Low maternal education<br>High parity<br>Emergency caesarean delivery<br>High risk patient risk | Unmet Gaps<br><br>Maternal mortality rates due to poor service delivery and socioeconomic factors<br><br>Gender constraints<br><br>Not identified during review | 1.9,208 deliveries were recorded during the period. 1/5 (20.5%) had no antenatal care before delivery, 79.5% had at least one antenatal care during pregnancy. 80.5% were normal deliveries, elective and emergency caesarean section rates were 3.1% and 11.5% respectively. 79 maternal deaths were recorded and 8,526 live births giving a maternal ratio of 927 maternal deaths per 100000 live births.<br>Low maternal education<br>2.High parity, emergency caesarean delivery<br>High risk patient risks were all independently shown to predict maternal mortality.<br><u>Recommendations</u><br>Multi sectorial approaches and focused political will are needed to improve delivery care and standards in Nigeria |

| | | | | | | |
|---|---|---|---|---|---|---|
| 55<br>Umoiyoho<br>et al 2010<br>Nigeria<br>Peer Reviewed<br>Journal Article | Title:<br><br>Perceptions among the Annang women of south-south Nigeria regarding antenatal healthcare information<br><br>Objectives:<br><br>Examine the attitude of pregnant women towards orthodox antenatal care and their understanding and implementation of care advice offered at clinics | Cross sectional village survey<br><br>Survey method from 2004-2005 on perception/attitudes and reasons for obtaining formal antenatal care.<br><br>Administered questionnaires on a total of 1560 women | *Reliability*<br>clear<br><br>*Internal validity:*<br>clear<br><br>*External validity*:<br>clear<br><br>*Limitations:*<br><br>Not stated in the publications | <u>Outcome measures</u><br><br>Reasons for obtaining formal antenatal care<br><br>Language of service provision<br><br><u>Determinants</u><br><br>Husband/parental pressure<br><br>To appear modern<br><br>Obtain information on health issues | Unmet Gaps<br><br>Not identified in review<br><br>Gender Constraints<br><br>Not identified in review | 1. Midwives play an important role since respondents in the study wanted midwives to be the ones to offer them health educations at the antenatal clinics.<br>2. Reasons for attending antenatal were to; Husband/parental pressure, To appear modern, Obtain information on health issues. 65.3% also wanted the local language in the area used during antenatal service education and sensitization sessions<br>3. There is need to review the content and the method of delivery of health education programs, in addition to assessing patients understanding periodically. staffs should develop appropriate systems for evaluating teaching and modify content for results |

| | | | | | | |
|---|---|---|---|---|---|---|
| 56<br>Ekott et al<br>2013<br>Nigeria<br>Peer Reviewed Journal Article | Title:<br><br>Perceptions of pregnant women about antenatal care in a cottage Hospital in Port Harcourt, Nigeria<br><br>Objectives:<br><br>To assess user satisfaction with antenatal care services and identify constraints at service point | Post service evaluation/follow up antenatal visits at a cottage hospital<br><br>Semi-structured interview questionnaire was used to collect information<br><br>400 questionnaires were analyzed using SPSS Ver. 15 | *Reliability*<br>clear<br><br>*Internal validity:*<br>clear<br><br>*External validity*:<br>clear<br><br>*Limitations*<br><br>Not stated in publication | <u>Outcome measures</u><br><br>Satisfaction from use<br><br>Service constraints/ Challenges<br><br>*<u>Determinants</u>*<br><br>Long waiting times<br><br>Unfriendly staff attitudes at some service points | Unmet Gaps<br><br>Not identified in review<br><br>Gender Constraints<br><br>Not identified in review | 1. Satisfaction was high; health (64%).<br>Education (95.8%), pharmaceutical services (92.3%), medical consultation (64%)<br><br>2.Constraints were delayed attention across service points(lab, drugs, consultation)<br><br>3. Women were generally satisfied. Determinants found in this study serve as barriers and should be addressed by stakeholders to improve utilization for women services |

| | | | | | | |
|---|---|---|---|---|---|---|
| 57<br>Nikiema et al<br>2012<br>Burkina Faso<br>Peer Reviewed<br>Journal Article | *Title:*<br>Measuring women perceived ability to overcome barriers to healthcare seeking in Burkina Faso<br><br>*Objectives:*<br>Create and validate a synthetic measures of women's access to healthcare from a publically available and possibly internationally population based survey | Survey data from DHS<br><br>Seven questionnaires from Burkina Faso 2003 DHS<br><br>Exploratory construct analysis (EFA) and confirmatory factor analysis (CFA) used to evaluate factorial structure and construct validity of index. | *Reliability*<br>clear<br><br>*Internal validity:*<br>clear<br><br>*External validity*:<br>clear<br><br>Limitations:<br>The seven construct to determine care seeking could have limited other unforeseen factors in the study.<br>The seven items also target curative needs to healthcare. It remains difficult on how non-curative needs such as contraceptive use and antenatal care can be captured by the index to ensure its appropriate measures | Outcome measures<br><br>Access to healthcare<br><br>Determinants<br>Getting permission.<br>Getting money.<br>Knowing where to go to seek care.<br>Distance to facility.<br>Having to take transportation.<br>Not wanting to go alone.<br>Concern that they may not be female staff. | Unmet Gaps<br><br>Not identified in review<br><br>Gender constraints<br><br>Need to ask for permission from husband before seeking healthcare is directly connected to social norms around marital relations in SSA | 1. Factors tested here as determinants showed positive associations which influence women seeking for healthcare. |

| | | | | | | |
|---|---|---|---|---|---|---|
| 58<br>Zere E et al<br>2012<br>Ghana<br>Peer Reviewed Journal Article | <u>Title:</u><br><br>Inequities in maternal and child health outcomes and interventions in Ghana<br><br>Objectives:<br>Examine the equity dimension of child and maternal health outcomes and interventions using Ghana as a case study | National household survey(GHDS 2008)<br><br>Population based weighted, regression based measures: slope of inequality and relative index of inequality<br><br>Analysis by STATA 10 | *Reliability*<br>clear<br><br>*Internal validity:*<br>clear<br><br>*External validity*:<br>clear<br><br>*Limitations:*<br><br>Not identified in review | <u>Outcome measures</u><br><br>Maternal and child health interventions ( Skilled delivery, Delivery in health facility, Use of modern contraceptives, Use of obstetric services<br><br><u>Determinants</u><br><br>Wealth of individual(derived from asset indices) | Unmet Gaps<br><br>Not identified in review<br><br>Gender Constraints<br><br>Not identified in review | 1.Skilled attendance at birth, place of delivery, caesarean delivery increase significantly among the wealthiest compared to the poor. publically funded delivery services tend to benefit the wealthiest rather than poor(castro-Leal et al 2000) as asserted also by this study<br><br>2. Pro-rich inequities in most of the maternal and child interventions in Ghana are wide spread and need to be addressed.<br><u>Recommendations</u><br>Policy should address both demand side and supply side factors of maternal services to improve maternal health outcomes |

| | | | | | | |
|---|---|---|---|---|---|---|
| 59<br>Ha, P.Y.<br>et al 2012<br>Ghana<br>Peer Reviewed Journal Article | Title:<br><br>Effects of socio-economic deprivation and health service utilization on Antepartum and Intrapertum Stillbirth<br><br>Objectives:<br><br>To examine associations between HH wealth and antepartum and intrapertum stillbirths.<br>To assess if any differences in risk were mediated by utilization of health services during pregnancy. | Prospective population based surveillance system involving all women of child bearing age and their babies in Ghana<br><br>Multivariable logistic regression | *Reliability*<br>clear<br><br>*Internal validity:*<br>clear<br><br>*External validity*:<br>clear<br><br>*Limitations:*<br><br>Recall bias by women | <u>Outcome measures</u><br><br>Risk of intrapartum and antepartum still birth<br><br>Health service utilization<br><br>Place of delivery<br><br><u>Determinants</u><br><br>Wealth of woman | Unmet Gaps<br>Not identified in review<br><br>Gender Constraints<br><br>Not identified in review | 1. Poor women had a high risk of intrapertum stillbirth in this population and the risk did not appear to be influenced by health service utilization. Socioeconomic status was NOT associated with intrapertum or antepartum stillbirth in univariable and multivariable analysis.<br>2. Pregnancy care was associated with significant reduction in antepartum stillbirths in study but NOT intrapartum. Poor women received low pregnancy care and delivered at home. Odds of delivering at district hospital was greater (intrapartum/antepartum) compared to home and health center. |

| | | | | | | |
|---|---|---|---|---|---|---|
| 60<br>Bove, R.M.<br>et al 2012<br>Mali<br>Peer Reviewed Journal Article | Title:<br>Women's health in urban Mali: Social predictors and health itineraries<br><br>Objectives:<br><br>Impact of social mediators on pregnancy and illness | Cross sectional and retrospective in design<br><br>Mixed qualitative and quantitative methods compared with demographic characteristics of 2001 Mali demographic health surveys<br><br>Analysis was done with STATA Version 11 | *Reliability*<br>clear<br><br>*Internal validity:*<br>clear<br><br>*External validity*:<br>clear<br><br>*Limitations:*<br>Data were obtained one decade ago, but still useful in measuring health needs across the population. | <u>Outcome measures</u><br><br>Prenatal care<br>Delivery characteristics<br>Use of postnatal care<br>Women knowledge of contraception<br><br><u>Determinants</u><br><br>Marital factors<br>Family type<br>Social factors (Social power score measure index)<br>Household wealth (Household wealth index measure) | Unmet Gaps<br><br>Not identified in review<br><br><br>Gender Constraints<br><br>Women households, marriages ,social networks and their ability to exert social power within these structures influence their general and reproductive health needs | 1. Women reported a fairly homogenous perinatal healthcare utilization, with 81% going for at least one antenatal prior to delivery.<br>2. Majority of visits (77%) cost1000 CFA or less and 85% were paid by subjects husbands. nearly all women delivered at facility (98%) accompanied by agnatic kin (mother-in-law) in 52% cases friends (15%), husbands (13%)<br>3. Broader socioeconomic and nominal values still exist which push women away from accessing RH services. Service providers must design ways to capture and reach poor resources women to improve their use of pre and postnatal care services. |

| | | | | | | |
|---|---|---|---|---|---|---|
| 61<br>Ononokpono D.N. et al<br>2013<br>Peer Review Journal Article | Title:<br>Contextual determinants of maternal health care service utilization in Nigeria<br><br>Objectives:<br><br>Relation of community factors to the use of antenatal care services<br><br>Weather community factors moderated the association between individual characteristics and antenatal care visits | Cross sectional survey<br>2008 NDHS<br><br>Stratified ,two stage cluster design sample<br><br>4 models were fitted and used against the dependent (ANC visits) | *Reliability*<br>clear<br>*Internal validity:*<br>clear<br>*External validity*:<br>clear<br><br>*Limitations*<br><br>Recall bias,<br>Design used cannot infer causality effects<br>Community variables were constructed by aggregating individual level characteristics at community level which could have resulted in making inferences at a higher level based on information from data collected at lower level.<br>Definition of "groups" is difficult in multi-level analysis | <u>Outcome measures</u><br><br>Number of ANC Visits<br>Community health deliveries<br><br><u>Determinants</u><br><br>Community factors (Mass media,<br>Wealth,<br>Education, joint decisions, religion | Unmet Gaps<br><br>Not identified in review<br><br><br><br>Gender Constraints<br><br>Not identified in review | 1. Findings indicated that community factors associated with ANC visits and also acted moderators of the association between individual factors and antenatal care visits.<br>2. The study showed a positive association between community hospital delivery and ANC attendance. Community poverty was negatively associated with ANC visits (suggest lack of community resources/autonomy).<br>Mass media,<br>education, employment HH wealth index was positive with ANC (4 or more visits). |

| | | | | | | |
|---|---|---|---|---|---|---|
| 62<br>Rajesh Kumar Rai et al<br>2012<br>Nigeria<br>Peer Review Journal Article | Title:<br>Utilization of Maternal Health care services among married adolescents women: Insights from the Nigeria Demographic and Health Survey,2008<br><br>Objectives:<br>Factors associated with selected maternity services; ANC visits, Safe delivery postnatal care | Data from 2008 NDHS for 2,434 young mother (15-19) years during the five years preceding the survey<br><br>Pearson chi-square test and binary logistic regression | *Reliability*<br>clear<br><br>*Internal validity:*<br>clear<br><br>*External validity*:<br>clear<br><br>*Limitations:*<br>*None* | Outcome measures<br><br>Standard antenatal visits(at least 4 times)<br>Safe delivery<br>Postnatal care<br><br>Determinants<br><br>Place of residence, women education, husband education, religion, social group, work status, mass media, wealth quartile, birth intervals, region | Unmet Gaps<br><br>Not identified in review<br><br>Gender Constraints<br><br>Not identified in review | 1.35.1%, 28.1% and 31.6% had at least 4 ante visits, safe delivery and postnatal care respectively. Wealthier women were more five times more likely to use compared to poorest adolescents.<br>2. Adolescents with 4 or more ANC visits were more likely to use safe delivery. The odds of postnatal care were higher among those adolescent women who safely delivered their last birth compared with those women who had not utilized safe delivery care. The finding of the study suggests that adolescent obstacles exist to use.<br>Recommendations<br>Content and service delivery strategy of maternity programs needs to be reviewed to cater for the poor, uneducated and marginalized social groups and residing in rural areas |

| | | | | | | |
|---|---|---|---|---|---|---|
| 63<br>Corroon, M. et al 2013<br>Nigeria<br>Journal Article | Title:<br>The role of gender empowerment on reproductive health outcomes in urban Nigeria<br><br>Objectives:<br><br>To assess if women empowerment is associated with family planning use and maternal health behaviors | Study uses baseline household survey data from the Measurements , Learning and Evaluation Project(MLE) for the Nigerian Urban Reproductive Health Initiative (NU-RHI)<br><br>Two stage sampling to select HH from each of the six cities<br><br>STATA Ver 12<br>Multivariate logistic regression used to test associations | *Reliability*<br>clear<br><br>*Internal validity:*<br>clear<br><br>*External validity*:<br>clear<br><br>*Limitations:*<br>Empowerment measures were adopted from south asia. Work needs to be done to validate the appropriateness in the African context.<br>Study was limited to women married and NOT those unmarried or living with a partner.<br>Cross sectional, hence difficult to associate causality of empowerment with RH outcomes | <u>Outcome measures</u><br><br>Current use of FP<br>Skilled attendant at birth<br>Place of delivery<br><br><u>Determinants</u><br>Economic freedom<br>Empowered decision making beliefs<br>Freedom from prohibition(religion, husbands)<br>Empowered domestic violence | Unmet Gaps<br><br>Not identified during review<br><br>Gender Constraints<br><br>Study reveals the importance of context specific relationship between gender issues and reproductive health behaviors in Urban Nigeria.<br>Lack of gender effects in addressing RH issues in different context. | 1. Gender empowerment plays a vital role in women likelihood to use FP or maternal health service.27.6% used FP across all cities. Regional variations however exist. Economic freedoms and empowered domestic violence predicted institutional delivery use in all cities.<br>2.Access to money, decision making ,freedom from prohibition and empowered domestic violence was also positively associated with skilled attendant at birth |

| Reference/Country of origin/ type of Publication Pub ref. | Title/Objectives of study | Study design/ Data collection /Analytical methods | Reliability Internal validity External validity | Outcomes measures /determinants Identified | Unmet Gaps/Gender constraints identified | Main Findings /conclusions/ policy recommendations |
|---|---|---|---|---|---|---|
| 64<br>Erim et al<br>2012<br>Nigeria<br>Original Article<br>Journal Article | Title:<br>A rapid assessment of the availability and use of Obstetric care in Nigerian Healthcare facilities<br><br>Objective:<br>Rapid assessment on facility quality in proving Obstetric care | Cross sectional survey of randomly selected Nigerian healthcare facilities<br><br>Convenient sample of women receiving maternal case were interviewed | *Reliability*<br>clear<br>*Internal validity:*<br>clear<br>*External validity*:<br>clear<br><br>Limitations:<br>1.Purpose not comprehensive assessment of facility quality.<br>2.Study design used cannot also be used in infer causality<br>3.Recall and survivor bias may have set in to affect the responses provided | <u>Outcome measures</u><br><br>Facility quality<br><br>Pregnancy outcomes<br><br>Facility delivery<br><br><u>Determinants</u><br>Infrastructure<br>Service provision<br>Level of care(primary, secondary and tertiary)<br>Staff available | Unmet Gaps<br>Not identified during review<br><br>Gender Constraints<br><br>Not identified during review | 1.only 40 %(N=121 facilities) primary care facilities provided 24 hour service for obstetric care<br>2. Half of facilities had a nurse/midwife available. lacked doctors, especially specialist obstetricians<br>3.about 4% of pregnancies observed ended up in stiibirth,6.6% as spontatous abortion whiles 2.4% were electively terminated<br>4Crude risk of reported still birth did not vary by place of birth or who conducted the birth. Maternal complications reported were fever, obstructed labour bleeding and eclampsia) |

| | | | | | | |
|---|---|---|---|---|---|---|
| 65<br>Okoli et al<br>2012<br>Nigeria<br>Journal article | Title:<br>Parental care and basic emergency obstetric care services provided at primary healthcare facilities in rural Nigeria<br><br>Objective:<br>To assess the availability of parental care and basic emergency obstetric care services at primary care level | Cross sectional descriptive design<br><br>Information obtained with an semi-structured interview method and from PHC service activity registers | *Reliability*<br>clear<br>*Internal validity:*<br>clear<br>*External validity*:<br>clear<br><br>Limitations:<br>Data was collected from PHC registers that is sometimes poorly recorded And lacking clearity<br>2.PHC facilities were selected using a set of criteria. results thus reflects what more patterns to PHC facilities | <u>Outcome measures</u><br>Facilities providing BEmOC<br><br>8 components of care satisfied<br><br><u>Determinants</u><br><br>Number of health staff<br>Poor infrastructure and logistics to meet prenatal needs | Unmet Gaps<br>Not identified during review<br><br><br>Gender Constraints<br>Not identified during review | 1.97% of 652 PHC facilities was providing some form of prenatal care<br>2. A large proportion (44%) of PHC facilities did not provide all components of prenatal care services<br><u>Recommendations</u><br>More commitment and resources to guarantee a good start in pregnancy will ultimately improve maternal and neonatal outcomes in poor settings |

| | | | | | | |
|---|---|---|---|---|---|---|
| 66<br>Storeng et al 2012<br>Burkina Faso<br>Original Article<br>Journal Article | Title:<br>Mortality after near –miss obstetric complications in Burkina Faso: medical, social and health –care factors<br><br>Objective:<br>Investigate mortality in women in Burkina Faso | Case control study on a main sample of 484 women-242 maternal deaths and 242 near misses and on a nested sample of 56 women | *Reliability*<br>clear<br>*Internal validity:*<br>clear<br>*External validity*:<br>clear<br><br>*Limitations:*<br>1. Income data was not taken. catastrophic expenditure was calculated based on a different study<br>2.Sample size was relatively small<br>3. Estimates were based on results that reach the health care system. (75.4% Hops which is high) | <u>Outcome measures</u><br><br>Obstetric maternal outcomes<br>Delay in seeking care<br>Delay in reaching a health facility<br>Delay in been provided with appropriate care<br><br><u>Determinants</u><br><br>Residence<br>Distance to facility(40km or more)<br>Education<br>Catastrophic expenditures | <u>Unmet Gaps</u><br>Not identified in review<br><br><br>Gender Constraints<br>Not identified in review | 1.Diagnosis and blood transfusion were two significant obstetric outcomes for women in study<br>2. High cost was associated with seeking for obstetric care<br>3.Delay factors are attributed to distance and sometimes cost implications<br>4. Wealthier households obtained reliable and fast care for obstetric needs compared to poor households<br>5. catastrophic spending had higher odds in eclampsia(OR:2.63;95% CI:1.44-4.83) and postpartum infection (OR:5.64;95%CI:2.51-12.65) |

| | | | | | | |
|---|---|---|---|---|---|---|
| 67<br>Filippi et al<br>2010<br>Benin<br>Journal Article | Title:<br>Effects of severe obstetric complications on women health and infant mortality in Benin<br><br>Objective:<br>To document impact of severe obstetric complications on post –partum health in mothers and mortality | Prospective cohort study contrasting post-partum health of three groups of women who gave | *Reliability*<br>clear<br>*Internal validity:*<br>clear<br>*External validity*:<br>clear<br><br>*Limitations*<br><br>*Not identified in review* | <u>Outcome measures</u><br><br>Delivery risk<br>Severe complications<br>Perinatal death<br><br><u>Determinants</u><br><br>Wealth<br>Age | <u>Unmet Gap</u><br><br>Not identified in review<br><br><u>Gender Constraints</u><br><br>Not identified in review | |

| | | | | | | |
|---|---|---|---|---|---|---|
| 68<br>Adanu RM<br>2010<br>Ghana<br>Peer Reviewd<br>Journal Article | Title:<br><br>Utilization of obstetric services in Ghana between 1999 and 2003<br><br>Objective:<br><br>To assess obstetric utilization levels in Ghana | Cross sectional study involving women attending antenatal care in health institution<br><br>GHDS 2003 Findings | *Reliability*<br>clear<br>*Internal validity:*<br>clear<br>*External validity*:<br>clear<br><br>Limitations:<br>Not identified in review | <u>Outcome measures</u><br><br>Obstetric utilization<br>Quality of obstetric care<br><br><u>Determinants</u><br><br>Women education<br>Health staff educational improvements<br>Health infrastructure | Unmet Gaps<br>Not identified in review<br><br>Gender Constraints<br>Not identified in review | 1. Even though 90% of pregnant women attend antenatal, only 43% deliver at health institutions.<br>2.National caesarean section rate of 3.7% reflects inadequate obstetric coverage<br><br><u>Recommendations</u><br>The health system needs to consider how to improve obstetric coverage by skilled attendants and study the reasons for inadequate use of delivery services in order to be able to achieve the target for maternal health set in MDGs. |

| | | | | | | |
|---|---|---|---|---|---|---|
| 69<br>Pirkle, C.M.<br>et al 2011<br>Mali<br>Original Article<br>Journal Article | Title:<br>Emergency Obstetric complications in a Rural Africa (Kayes, Mali): The link between travel time and In-Hospital Maternal Mortality<br><br>Objective:<br><br>To asses emergency obstetric complications in rural setting | Descriptive case series<br><br>Matched case control design | *Reliability*<br>clear<br>*Internal validity:*<br>clear<br>*External validity*:<br>clear<br><br>*Limitations*<br>Inadequate sample size to detect significance for travel times of 2-3 hrs from the hospital | <u>Outcome measures</u><br><br>Obstetric complications<br>Maternal death<br>Spatial access<br><br><u>Determinants</u><br>Time travel(more than 4 hrs) | Unmet Gaps<br>Not identified in review<br><br><br>Gender Constraints<br><br>Not identified in review | 1.2359 women were treated for obstetric complications(Jan 2005-Dec.2007)<br>2. survival rates was known for 2234;47 resulted in maternal deaths, yielding case fatality rate of 2.1%<br>3.prmary diagnosis for deaths were pre-eclampsia (n=13,6.5%), hemorrhage (n=13,4.3%),indirect causes(n=7,4.7%),uterine rapture(n=6,19.4%),other direct cause(n=3,0.8%), Obstructed labor (n=3, 0.4%), infection (n=1, 5.5%) and miscarriage/abortion (n=1, 0.7%).<br>4.Case fatality increased with time travel<br><u>Recommendations</u><br>Supports the hypotheses that even women who reach EmOC, considerable travel times increase chances of maternal mortality.<br>Efforts at making services closer to bring good health outcomes |

| | | | | | | |
|---|---|---|---|---|---|---|
| 70<br>Ijadunola, K.T. et al 2010<br>Nigeria<br>Original Article<br>Journal Article | Title:<br>New paradigm old thinking: the case for emergency obstetric care in the prevention of maternal mortality in Nigeria<br><br>Objective:<br>Assessed the knowledge of maternity unit operatives at the primary and secondary level care on obstetric care | Descriptive design, exploring perception, knowledge and practices of maternity operatives. | *Reliability*<br>clear<br>*Internal validity:*<br>clear<br>*External validity*:<br>clear | Outcome measures<br><br>Knowledge of health care operatives of EmOC<br><br>Determinants<br>Awareness of LSS<br>Number trained on LSS | Unmet Gaps<br><br>Gender Constraints | Maternilty care operatives at the level of primary and secondary care had poor knowledge of EmOC |

| | | | | | | |
|---|---|---|---|---|---|---|
| 71<br>Ogu, R. et al<br>2012<br>Nigeria<br>Original Article<br>Journal Article | Title:<br>Outcome of an intervention to improve the quality of private sector provision of post abortion care in Northern Nigeria<br><br>Objective:<br>To assess intervention outcomes for post abortion care in Nigeria | Intervention case study | *Reliability*<br>clear<br>*Internal validity:*<br>clear<br>*External validity*:<br>clear | <u>Outcome measures</u><br><br>Reasons for seeking abortion and post abortion services<br><br><u>Determinants</u><br><br>Cost | Unmet Gaps<br>Not identified in review<br><br>Gender Constraints<br>Not identified in review | 1.women stated reasons for seeking abortin care included; continue education, poor state of health, poor economic circumstances, pregnancy due to rape, fear of parents, partner refusal f pregnancy still lactating |

| | | | | | | |
|---|---|---|---|---|---|---|
| 72<br>Hill, Z.E. et al<br>2009<br>Ghana<br>Journal Article | Title:<br>The context of Informal Abortions in Rural Ghana<br><br>Objectives:<br>Identify areas in Ghana which should be considered for abortion related startegies | Descriptive cross sectional<br>Population based surveillance between September, 2005-March, 2006<br>Two datasets (Verbal postmortems and pregnancy narratives)<br><br>Focus group discussions were also employed | *Reliability*<br>clear<br>*Internal validity:*<br>clear<br>*External validity*:<br>clear<br><br>Limitations | <u>Outcome measures</u><br><br>Unsafe abortion methods options<br><br><u>Determinants</u><br><br>High cost of clandestine clinic abortion<br>Confidentiality<br>Access to use of service | Unmet Gaps<br>Not identified in study<br><br>Gender constraints<br>Not identified in study | 1.Most frequently used method options are; use of sugar-related substances, herbal mixtures, analgesics, ground bottles, hormonal preparations<br>2. Study suggests rural women are wide aware of the several unsafe options available for them when in need for abortion. Due to high cost for clandestine abortion services, most resort to these unsafe modes for their "survival''<br>*<u>Recommendations</u>*<br>Services must be accessible, affordable, and confidential<br>Discourse with communities and health workers is needed to break the public silence about abortion |

| | | | | | | |
|---|---|---|---|---|---|---|
| 73<br>Konney O et al<br>2009<br>Ghana<br>Journal Article | Title:<br>Attitude and women with Abortion related complications towards provision of safe abortion services in Ghana<br><br>Objectives:<br>Determine attitudes and perceptions of women with abortion-related complications towards provision of safe abortion services | Cross sectional survey of women at KATH<br>SPSS for analysis | *Reliability*<br>clear<br>*Internal validity:*<br>clear<br>*External validity*:<br>clear<br><br>*Limitations*<br>*Not identified in review* | Outcome measures<br><br>Abortion complications<br>Knowledge of abortion laws<br><br>Determinants<br>Family union status<br><br>Legal knowledge of abortion<br><br>Stigma associated with unmarried pregnancy | Unmet Gaps<br>Not identified in review<br><br>Gender Constraints<br>Not identified in review | 1.A high number (92% N= 296) did know abortion law existed.<br>Those who reported induced abortions were more likely to have had formal education and more likely to be unmarried(spontaneous abortions)<br>Burden of abortion related cases in Ghana averages 38.8%-40.7% in two previous studies in Ghana |

| | | | | | | |
|---|---|---|---|---|---|---|
| 74<br>Abiodun, O.M.<br>et al 2013<br>Nigeria<br>Journal Article | Title:<br>Complications of unsafe abortion in South West Nigeria: a review of 96 cases<br><br>Objective:<br>To determine the sociodemographic characteristics and pattern of complications present in patients managed for unsafe abortion | Hospital records and other secondary data reviewed and analyzed | *Reliability*<br>clear<br>*Internal validity:*<br>clear<br>*External validity*:<br>clear<br><br>*Limitations*<br><br>*Not identified in review* | Outcome measures<br><br>Complications of abortion<br><br>Gestational period before abortion<br><br>Determinants<br>Status<br>(student, not)<br>Family union<br>Age | Unmet Gaps<br>Not identified in review<br><br>Gender Constraints<br>Not identified in review | 1.Majority were less than 25 years(60.4%).74.0% were students whiles 81.3% were not married<br>2.47.9% were performed in the second trimester of gestation whiles 21(21.9%) were performed within 8 weeks of gestation<br>3.Complications such as Sepsis was common(79.2%) whiles uterine perforation was present in 12.5% of women<br>4.Unsafe abortion from study accounted for 30.8% of maternal deaths during the period<br>Recommendations<br>Adolescent reproductive interventions must target young nulliparous women and girls who need informed information to avoid unwanted pregnancies or use safe abortion services if the need be. |

| | | | | | | |
|---|---|---|---|---|---|---|
| 75<br>Ibrahim, I.A.<br>et al 2012<br>Nigeria<br>Journal Articles | Title:<br>Sociodemographic determinants of complicated unsafe abortions in a semi-urban Nigerian Town: a four year review<br><br>Objective:<br>Aim was determine the sociodemographic factors involved in complicated unsafe abortions | Four year retrospective evaluation of unsafe complicated abortions | *Reliability*<br>clear<br>*Internal validity:*<br>clear<br>*External validity*:<br>clear<br><br><br>*Limitations*<br>Not identified in review | Outcome measures<br><br>Unsafe complicated abortions<br>Maternal mortality rates<br><br>Determinants<br><br>Unfavorable sociodemographic factors<br>Lack of knowledge of abortion laws | Unmet Gaps<br>Not identified in review<br><br><br>Gender Constraints<br><br>Not identified in review | 1. Incidence of unsafe complicated abortions was 4.10% of total deliveries and contributed 14.0% of gynecological admissions.<br>2.There were 55.45% of patients who were nulliparae,60.32% were unemployed and 69.80% were unmarried. A total of 87.30% had never used any form of contraception<br>3.Abortion mortality was 256/100000 deliveries and the case fatality was 4.76%.17.64% of maternal deaths during the study period. Common identified cause of death was Septicemia(66.66%)<br>Recommendations<br>Measures at addressing unsafe abortion and its complications should be multi-targeted approaches |

| | | | | | | |
|---|---|---|---|---|---|---|
| 76<br>Payne, C.M.<br>et al 2013<br>Ghana<br>Journal Article | Title:<br>Why women are dying from unsafe abortion : narratives of Ghanaian abortion providers<br><br>Objectives:<br>To assess why women dye from abortion related issues | Cross sectional survey | *Reliability*<br>clear<br>*Internal validity:*<br>clear<br>*External validity*:<br>clear<br><br>*Limitations*<br>Not identified in review | Outcome measures<br><br>Reasons for Unsafe seeking options<br><br>Determinants<br><br>Accessibility<br>Cultural acceptability<br>Inadequate Physicians to provide care<br>Financial constraints<br>Lack of adequate knowledge on abortion laws and regulations<br>Stigma of seeking care | Unmet Gaps<br>Not identified in review<br><br>Gender Constraints<br>Not identified in review | 1. Interview findings indicate majority of unsafe abortions complications arise in setting of clandestine or self-induced second trimester attempts, suggesting that training greater number of physicians to perform second trimester abortions is key.<br>2. Stigma, financial cost, low knowledge of abortion laws are some reasons why people seek unsafe abortion care services<br>Recommendations<br>Plans to address maternal mortality must address the multiple dimensions which influence the practice and utilization of safe abortion ,especially the second trimester |

*Clear attributes of reliability, internal (content) and external (face) validity indicates that measures estimates are appropriately measured and provide easy understanding for readers.

** Unclear attributes of reliability, internal (content) and external (face) validity indicates that measures estimates are not appropriately measured and are not easily understood by readers.
